# Tracing embodied CO_2_ emissions and drivers in China’s financial industry under inter-provincial trade

**DOI:** 10.1038/s41598-024-79833-x

**Published:** 2024-11-19

**Authors:** Donghua Xiao, Wenhui Guo, Xincong Liu, Yi Zheng, Hao Gong, Chuan Wang

**Affiliations:** 1 School of Management, Xihua University, 611000 Chengdu, People’s Republic of China; 2 Research Institute of International Economics and Management, Xihua University, 611000 Chengdu, People’s Republic of China; 3 School of Architecture and Civil Engineering, Chengdu University, 610106 Chengdu, People’s Republic of China

**Keywords:** CO_2_ emissions, Financial sector, Interprovincial trade, Embodied emissions, Multiregional input–output, Climate-change policy, Energy and society, Environmental economics, Sustainability

## Abstract

With the establishment of “Dual Carbon” targets and industrial restructuring in China, the transition from the secondary industry to the tertiary industry has facilitated the rapid development of the financial sector. However, the significant CO_2_ emissions embodied within inter-provincial trade result in carbon leakage, posing challenges in assigning equitable carbon reduction responsibilities to the financial sectors across the 31 provinces of China. This study establishes a framework for evaluating CO_2_ emissions of financial sectors through 134 samples of 60 listed financial enterprises in the 31 provinces, tracking the embodied CO_2_ emissions within inter-provincial trade by using a multiregional input–output approach. The results reveal that the total CO_2_ emissions of the financial sector in China surged from 4.591 to 12.423 Tg CO_2_-eq between 2012 and 2020. The regions with the highest annual net CO_2_ emissions are Anhui (0.244 Tg), Zhejiang (0.242 Tg), and Henan (0.211 Tg). The key factors influencing net CO_2_ emissions are in the following order of importance: net CO_2_ density, per capita added value of service industry, the proportion of finances in service industries, and population size. Based on the findings, this study provides policy implications: reducing net carbon intensity, enacting tailored carbon tax policies based on embodied CO_2_ emissions, and fostering interdepartmental collaboration to address the impact of carbon leakage.

## Introduction

China is ambitious to announce the “Dual Carbon” strategy at the 75^th^ General Assembly of the United Nations^1^, aiming to achieve the peak of carbon dioxide emissions by 2030 and achieve carbon neutrality by 2060^2^. As the world’s largest energy consumer and CO_2_ emitter^3,4^, China focuses on developing extremely important policies for optimizing and upgrading the industrial structure by developing low-carbon industries, especially the service industry^5^. The financial industry, an important sector within service industries contributing significantly to the added value of over 15.15%^3^, was regarded as the ‘zero’ CO_2_ emissions^6^ and rapidly developed in the 10^th^ to 13^th^ Five Year Plan (2001–2020) of China, with the increasing value added over 30 times^7^. However, it is imperative to take cognizance of the CO_2_ emissions from the Chinese financial sector, due to its large-scale material consumption, labor, and service inputs, which produce indirect CO_2_ emissions during its life cycles^8^. Currently, these indirect CO_2_ emissions have not yet been fully revealed.

Then, compared with the other sectors of the service industry, the finance sector is an industry with frequent trade among all provinces, where the producers and consumers are often not in the same province. The trade activities among different provinces lead to an unequal distribution of responsibilities for CO_2_ emission reduction. Usually, the provinces of the service supplier or producer have a larger responsibility rather than that of the final consumers^9^. Therein, demand for external finance promotes the development of the financial industry^10^, leading to inequality of dividing responsibility when using production-based accounting of the CO_2_ emissions. In fact, a large part of CO_2_ emissions in the financial sector are caused by the carbon leakage embodied in the inter-provincial trade^11^. Therefore, provinces located downstream of the financial industry chain and receiving the financial service, need to demonstrate more embodied CO_2_ emissions and bear the corresponding emissions reduction responsibility.

**Fig. 1:**
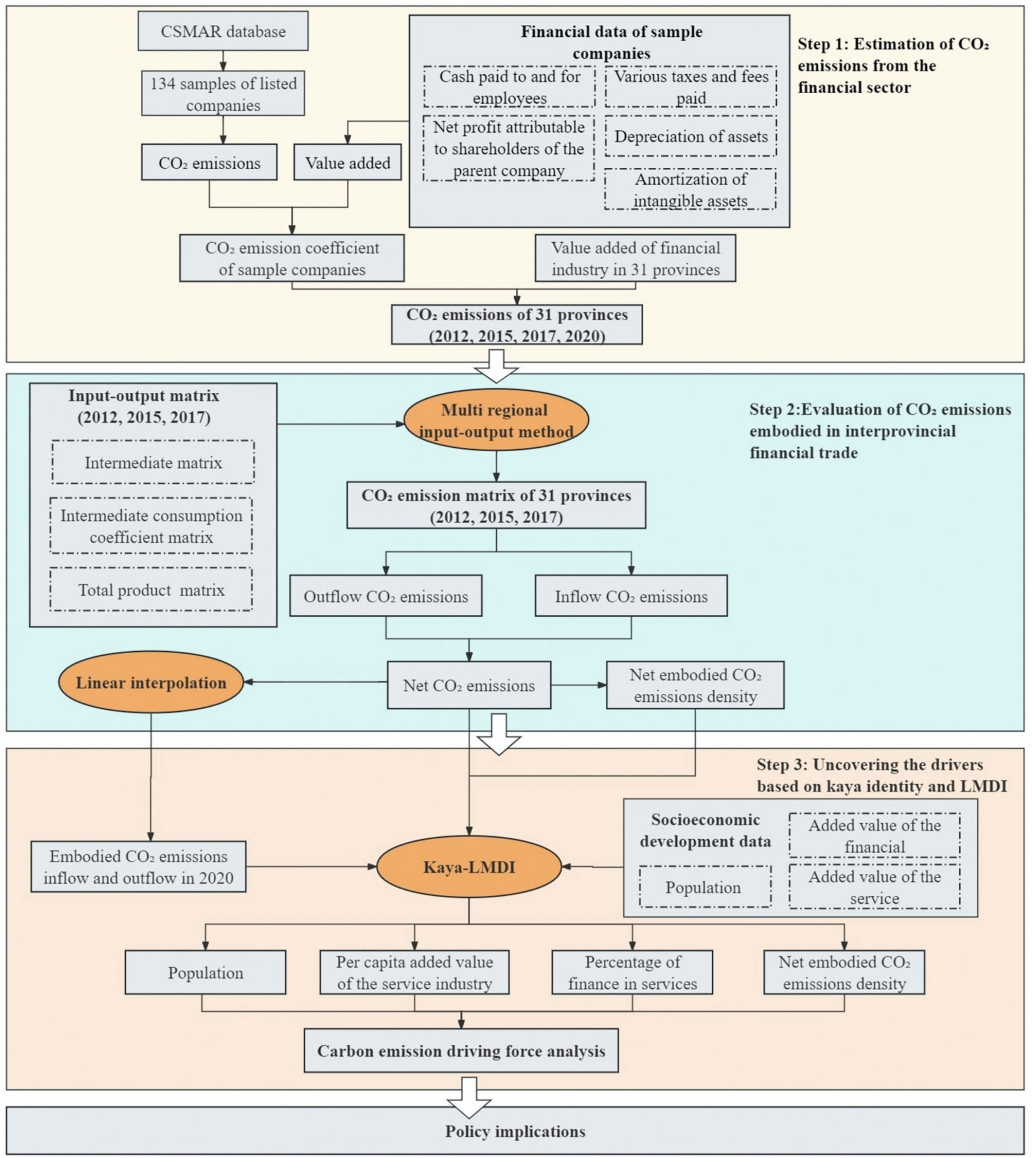
Roadmap of this study.

Although the Chinese government has been aware of the CO_2_ emissions of financial enterprises and has announced the policy in accounting for the CO_2_ emissions of financial enterprises. For example, in 2024, the Ministry of Ecology and Environment of China (MEE of China) announced a policy that promotes financial institutions to conduct life cycle emissions accounting^9^. Besides, the People’s Bank of China (PBC) released the “Technical Guidelines for Carbon Accounting in Financial Institutions (Trial)” to assist financial institutions in accounting for their own CO_2_ emissions and reduction amounts^10^. However, these policies are formulated primarily based on administrative regional divisions, lacking a profound consideration of regional interconnectedness and the spillover effects of financial sector CO_2_ emissions. In this context, this study establishes a framework to evaluate the CO_2_ emissions in the 31 provinces of China, tracing the embodied CO_2_ emissions within the inter-provincial trade, and aiming to provide specific policy implications for policymakers of 31 provinces. The rest of this study is structured as follows: Section 2 reviews the latest literature about the definition and evaluations of embodied CO_2_ emissions. Section 3 describes the establishment of the evaluated framework and data sources. Section 4 presents the main results. Based on the results, Section 5 discusses the trend and driving forces of the embodied CO_2_ emissions. Finally, we conclude the results and integrate the results and discussion into the policy design in 31 provinces. The road map of this study is shown in Fig. 1.

## Related literature review

### Sources of financial sector CO_2_ emissions

In previous studies, the service industries including the financial industry were usually regarded as the low-carbon industries^12,13^. However, due to the large scale of the service industry in China, the CO_2_ emissions it generates cannot be ignored, and it has even become the second-largest industry in terms of carbon footprint^14,15^. The report of the Intergovernmental Panel on Climate Change (IPCC) in 2019 suggested the sources of CO_2_ emissions in the service industries mainly from the consumption of energy, such as electricity and fossil fuel^16^. Based on the IPCC report, ISO 14,044, and 14,067^70^, the Chinese government has released the national standard “Greenhouse Gas Product Carbon Footprint Quantitative Requirements and Guidelines” (GB/T 24,067). In 2023, to achieve the “Dual Carbon” strategy, the MEE of China announced a policy that promotes financial institutions to conduct life cycle emissions accounting^17^. In 2024, the PBC released the “Technical Guidelines for Carbon Accounting in Financial Institutions (Trial)” to assist financial institutions in accounting for their own CO_2_ emissions and reduction amounts^18^. Following the guidelines, the life cycle emissions of financial institutions mainly include energy consumption (such as electricity and fossil fuel), raw material consumption (such as paper, office supplies, and labor protection appliances), and natural resource consumption (such as water use).

Many previous studies have revealed the CO_2_ emissions of the financial industry. For example, CO_2_ emissions of Chinese financial institutions from 2000 to 2009 were evaluated indicating that the enterprises of financial intermediation and banks were the largest emitters^19^. By collecting data from listed companies from 2007 to 2017, the CO_2_ emissions of various industries in China were calculated, with the CO_2_ emissions of the financial industry mainly concentrated in the southeast coastal region of China^20^. However, many previous studies treated financial enterprises as producers of CO_2_ without considering the promotional effect of financial trade on the CO_2_ emissions of these enterprises. Attributing all the CO_2_ emissions to the producers is unfair^21,22^. In fact, large amounts of CO_2_ emissions are driven by consumers from trade^23^, leading to carbon leakage^24^. Therefore, evaluating accurate CO_2_ emissions embodied in the trade is a need for quantifying the CO_2_ emission reduction responsibilities between producers and consumers more equally^25^.

### Evaluation of CO_2_ emissions at industrial scale

Many studies have accounted for CO_2_ emissions from several industries^71^. For example, comprehensively employing the emission factor method, a network approach that simulates cross-regional electricity flow and an environmentally extended input–output model, study^26^ provided systematic electricity-related CO_2_ emissions accounting for regional electricity-related CO_2_ emissions under multi-scope using the case of Shanghai from 2007 to 2012. Industry-level total CO_2_ emission factor performance for 25 cities was evaluated in the Yangtze River Basin during 2007–2016 through the distance function and regression based on the inventory method^27^. Based on data envelopment analysis and linear regression methods, study^28^ evaluated the CO_2_ emission efficiency and reduction cost in 30 provinces of China during 1996–2012. However, these linear regression-based methods might have uncertainty when evaluating the CO_2_ emissions in various industries and regions, due to the different carbon densities. To address this, linear regression combined with sampling can reduce the uncertainty of CO_2_ emissions in specific industries, such as the financial sector. For example, combined with sampling on tourism in Chengdu during 1991–2018, the regression line was established to evaluate the CO_2_ emissions in the tourism industry^29^. Regression combined with GIS sampling was used to evaluate the CO_2_ emissions in the south of the Netherlands on the city scale^30^. By constructing a multivariate regression and hierarchical regression model, study^31^ proposed a new method of estimating city-level CO_2_ emissions using nighttime light combined with the city’s socioeconomic attributes fitting coefficient for each city and improved the methods of estimating city-level CO_2_ emissions based on nighttime light. Using a non-radial, non-directional relaxation measure-based directional distance function model, the CO_2_ emission efficiency of 284 cities over the period from 2006 to 2020 was assessed^32^.

### Embodied CO_2_ emissions flow between industries and regions

In previous studies, the accounting of financial industry CO_2_ emissions has been predominantly from a production-oriented perspective, attributing emissions primarily to producers. However, this approach neglects the driving force of consumption in CO_2_ emissions, resulting in carbon leakage from the trade^33^. In fact, consumption is the ultimate purpose of production and the root cause of CO_2_ emissions^34^. Therefore, consumption-based embodied CO_2_ accounting can provide incentives for emission reduction in the financial industry, helping to mitigate the risks of carbon leakage^6^.

In terms of the evaluating scales, the assessments on quantifying embodied CO_2_ emissions are predominantly conducted at a macro-scale. Many studies have assessed different countries or regions^35,36^. Then, from the sights of the industrial scale, many studies have evaluated the embodied CO_2_ emissions of the labor-, energy- and resource-intensive industries with large direct CO_2_ emissions, such as construction^37^, agriculture^38^, and manufacturing^39^. However, few studies focus on industries with relatively low direct CO_2_ emissions but with a large trade volume, such as the financial sector.

In terms of evaluating methods, Multi-Regional Input–Output (MRIO), first proposed by Isard^40^, is widely used for consumption-based CO_2_ emissions accounting^41^. For example, based on the dataset of Beijing, study^42^ used MRIO to identify the net backward linkage as the major contributor to CO_2_ emissions of the financial industry. Therefore, MRIO can offer a comprehensive sight to quantify the responsibility for reducing emissions of the provinces from a consumer-oriented perspective, providing the basis for formatting specific region policies^43^.

### Methods of exploring driving forces

In previous studies, the method of impact, population, asset, and technology (IPAT) is widely used in driving force decomposition for identifying the relationship between environmental pollution and social development^44^. Further, based on the IPAT, Kaya identity was developed to identify the relationship between CO_2_ emissions and socioeconomic development^45^. Compared with IPAT, Kaya identity has little limitation on the choice of the types of driving forces, however, there is also a lack of quantification on these driving forces. The widely used methods for quantifying the driving forces are structural decomposition analysis (SDA)^41^ and index decomposition analysis (IDA)^46,47^. Therein, the SDA model focuses on the evaluation in large-scale regions. While the IDA model focuses more on specific industries, without the demand for long-term monitoring data. For example, study^48^ employed the IDA approach to quantify and decompose CO_2_ emissions inequality, utilizing data spanning from 2006 to 2021 for 89 cities. Logarithmic Mean Divisia Index (LMDI), one of the IDA-based methods, was widely used to explore the driving forces of environmental pollution, due to its properties of facilitating the interpretation and “zero” values in its decomposition^49^. Therefore, Kaya-LMDI is widely used for exploring the driving forces of CO_2_ emissions in recent studies CO_2_ emissions. For example, based on the Kaya-LMDI, study^50^ decomposed the CO_2_ emissions of the construction industry in Shandong, indicating that economic and service levels were the main contributors to the CO_2_ emissions of the construction industry. Through the Kaya-LMDI, it was found that economic and demographic factors were the main drivers promoting the increase in construction waste generation in the EU and China^51^. Therefore, utilizing the Kaya-LMDI method enables the identification of the pivotal drivers in the net CO_2_ emissions across diverse regions, offering a pivotal strategy for optimizing CO_2_ emissions in China’s financial industry.

### Contributions of this study

After reviewing the above literature, we found some research gaps that can be concluded as follows: first, the government and the public have recognized the CO_2_ emissions of large-scale financial enterprises in China, but there is currently no comprehensive framework for accounting for CO_2_ emissions; second, attributing all CO_2_ emissions to the financial institution is unfair, for the carbon leakage from the trade among 31 provinces; third, the drivers of CO_2_ emissions in the financial sectors include multiple factors such as social, economic, population, and carbon intensity, and ignoring these factors may lead to difficulty in policy formulation. To address these research gaps, this study established a comprehensive framework for evaluating CO_2_ emissions from the financial sector. Unlike previous studies, this framework offers more accurate assessments of CO_2_ emissions by integrating 134 samples of 60 listed financial enterprises which covered over 87% of the total market capitalization of listed financial firms in China. Then, we evaluated the embodied CO_2_ emissions induced by demand through interprovincial trade and uncovered the driving forces and factors contributing to embodied CO_2_ emissions through the IDA-based approach. Overall, this study provides valuable insights for policymakers in a leading financial nation (such as China), which can help formulate systematic strategies for CO_2_ emissions reduction in the financial sector.

## Methods

### Low carbon development of the financial sector in China

**Fig. 2:**
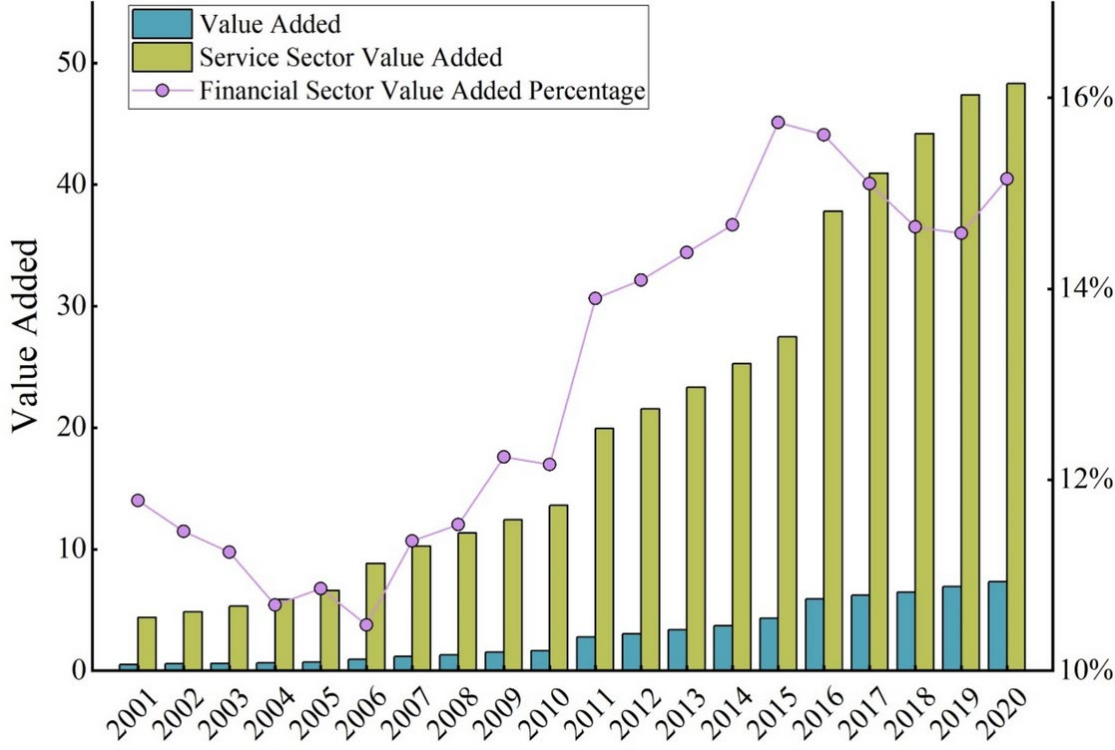
Value added of China’s financial and services sectors.

From 2001 to 2020, the percentage of China’s financial sector in the service sector increased from 10.48% to 15.74%. As shown in Fig. 2, the value added of China’s financial sector has increased from 5.18 × 10^11^ yuan to 73.15 × 10^11^ yuan. And the value added of China’s service sector has increased from 43.99 × 10^11^ yuan to 482.97 × 10^11^ yuan. The development of China’s financial industry exhibits notable regional disparities^52^. Specifically, the eastern coastal regions, such as Jiangsu, Shanghai, and Zhejiang, boast relatively high financial development levels due to their advantageous geographical location and economic foundation. Besides, the northwestern region (e.g., Xizang, Qinghai, and Ningxia) and the northeastern region (e.g., Jilin, Heilongjiang) lag in financial development. These disparities in financial development complicate the allocation of CO_2_ emission responsibilities and the setting of emission reduction targets, thereby increasing the difficulty of addressing climate change.

In 2010–2015, referring to the IPCC Guidelines’ accounting methods, China’s National Development and Reform Commission (NDRC) released the "Provincial Greenhouse Gas Inventory Compilation Guidelines (Trial)" ^53,54^ for CO_2_ emissions accounting in different industries, incorporating the financial sector. In 2019, the China Banking and Insurance Regulatory Commission released the “Green Credit Project Energy Savings and Emission Reductions Calculation Guidelines”, offering clear methodologies and benchmarks for financial institutions to evaluate the carbon benefits of green credit projects and promoting innovation in green financial products^55^. In 2020, the Partnership for Carbon Accounting Financials (PCAF) released the “Global GHG Accounting and Reporting Standard for the Financial Industry”, a framework aimed at guiding financial enterprises in accounting for and disclosing Scope 3 carbon emissions^56^. In 2021, the PBC released the “Guidelines for Environmental Information Disclosure by Financial Institutions”^57^ and the “Operational Manual for Environmental Information Disclosure by Banking Financial Institutions (Trial)”^58^, which set clear disclosure requirements for CO_2_ accounting in financial institutions’ investment and financing activities. The subsequent release of the “Technical Guidelines for Carbon Accounting in Financial Institutions (Trial)”^59^ provided specific technical guidance for carbon accounting in scope 3 activities, promoting pilot CO_2_ accounting projects in financial institutions. In 2024, the PBC, along with six other departments, jointly issued the “Guiding Opinions on Further Strengthening Financial Support for Green and Low-Carbon Development”^60^. Currently, the 31 provinces have been conducting CO_2_ emissions accounting and formulating emission reduction plans for the financial sector.

### Estimation of CO_2_ emissions from the financial sector

In consideration of the lack of CO_2_ emission data disclosure in China’s financial industry, we used the estimation method to determine the total CO_2_ emissions of China’s financial industry. Firstly, we collected 134 greenhouse gas emission samples from the 60 financial enterprises in China as estimation samples, which were calculated by the IPCC Guidelines for National Greenhouse Gas Inventories and General Rules for Calculation of the Comprehensive Energy Consumption of China. Given the significant developmental disparities across different regions in China, we categorize the provinces where the samples are located into 7 regions: Northeast, North China, Central China, South China, East China, Northwest, and Southwest. However, due to the limitation of a few sample numbers in the Northeast, Northwest, and Southwest regions, these areas were integrated into a category labeled as “Other Regions”. The total national CO_2_ emissions (*TCE*) can be expressed as follows:

1 $$TCE=\sum_{m=1}^{31}{TCE}_{m}$$

where, ${TCE}_{m}$ is the total CO_2_ emissions of *m*th province. ${TCE}_{m}$ is calculated from the data of listed companies in the *m*th province, including the CO_2_ emissions of electricity, fossil energy consumption, input materials, labor, and water consumption. The estimation process is as follows:

2 $${TCE}_{\text{m}}={\xi }_{i}\times FV{A}_{m}$$

3 $${\xi }_{i}=\frac{{\sum }_{j=1}^{{n}_{i}}{\eta }_{ij}}{{n}_{i}}$$

4 $${\eta }_{ij}=\frac{{E}_{ij}}{V{A}_{ij}}$$

5 $$V{A}_{ij}={CPE}_{ij}+{NP}_{ij}+{TF}_{ij}+{DA}_{ij}+{AI}_{ij}$$

where, ${\xi }_{i}$ represents the CO_2_ intensity of the *i*th region that the *m*th province belongs to. And $FV{A}_{i}$ is the financial value added of *i*th region. ${\eta }_{ij}$ is the value-added emission factor of the *j*th company in the *i*th region. $V{A}_{ij}$ is the value added of the *j*th company in the *i*th region. The added value of an enterprise is calculated through the income-based output approach, encompassing cash paid to and for employees (*CPE*), net profit attributable to shareholders of the parent company (*NP*), various taxes and fees paid (*TF*), depreciation of assets (*DA*), and amortization of intangible assets (*AI*). The depreciation component includes depreciation of fixed assets, depletion of oil and gas assets, and depreciation of productive biological assets. The detailed value added of the financial industry of 31 provinces is provided in Table S1.

### Evaluation of CO_2_ emissions embodied in interprovincial trade

Based on the total CO_2_ emissions of 31 provinces in China, we utilized the MRIO method to evaluate the inflow and outflow CO_2_ emissions of the financial sector among 31 provinces in China, to explore the net embodied CO_2_ emissions from the financial trade. Due to the limitations of the research focus within China, we adopted the non-competitive import assumption, signifying the exclusion of international imports from the MRIO table. The MRIO table reflects the relationship among the total output, intermediate requirements, and final demand in a region, which can be expressed as follows:

6 $$\left[\begin{array}{c}{X}^{1}\\ {X}^{2}\\ {X}^{3}\\ \vdots \\ {X}^{r}\end{array}\right]=\left[\begin{array}{ccccc}{A}^{11}& {A}^{12}& {A}^{13}& \cdots & {A}^{1n}\\ {A}^{21}& {A}^{22}& {A}^{23}& \cdots & {A}^{2n}\\ {A}^{31}& {A}^{32}& {A}^{33}& \cdots & {A}^{3n}\\ \vdots & \vdots & \vdots & \ddots & \vdots \\ {A}^{n1}& {A}^{n2}& {A}^{n3}& \cdots & {A}^{nn}\end{array}\right]\left[\begin{array}{c}{X}^{1}\\ {X}^{2}\\ {X}^{3}\\ \vdots \\ {X}^{r}\end{array}\right]+\left[\begin{array}{c}\sum_{s} {Y}^{1s}\\ \sum_{s} {Y}^{2s}\\ \sum_{s} {Y}^{3s}\\ \vdots \\ \sum_{s} {Y}^{ns}\end{array}\right]$$

where *X*^1^ to *X*^*r*^ represents the column vector of the total output in region 1 to r. *A*^*r,s*^ represents the intermediate requirements, showing the input in region r required to produce one unit of output in region *s*. *A*^*r,s*^ is composed of the sub-requirements of every sector (${A}_{i,j}^{r,s}$), which shows the sub-requirements of the *i*th sector in region r required to the produce *j*th sector in region *s*. *Y*^*r,s*^ is the final demand in region s which is provided by region *r*. The Eq. (6) can be shown a simplification by Eq. (7). Based on Eq. (7), the total output can be expressed by Eq. (8):

7 $$X=AX+Y$$

8 $$X={(I-A)}^{-1}\times Y$$

where *X*,* A*, and* Y* represent the matrix of the total output, intermediate requirements, and final demand respectively. *I* is the identity matrix, and *(I-A)*^*-*1^ is the Leontief inverse matrix.

This study aims to explore the embodied CO_2_ of the financial sector; thus, it should establish a direct CO_2_ emissions intensity vector to expand the MRIO model, as follows:

9 $$CE=[{CE}^{1},{CE}^{2},\dots \dots {CE}^{n}]$$

where *CE* is the direct CO_2_ emissions intensity vector, which is composed of direct CO_2_ emissions from region 1 to n (*CE*^1^ to *CE*^n^). Based on Eq. (9), the total CO_2_ emissions matrix can be expressed as:

10 $$\widehat{TCE}=\left[\begin{array}{ccccc}{CE}^{1}& 0& 0& \cdots & 0\\ 0& {CE}^{2}& 0& \cdots & 0\\ 0& 0& {CE}^{3}& \cdots & 0\\ \vdots & \vdots & \vdots & \ddots & \vdots \\ 0& 0& 0& \cdots & {CE}^{n}\end{array}\right]$$

where the $\widehat{TCE}$ is the diagonalization matrix of vector *CE.* In the $\widehat{TCE}$, it highlights that the CO_2_ emissions would be only allocated to the financial sector of every province, and other sectors share zero of the CO_2_ emissions. Thus, the embodied CO_2_ emissions can be calculated as follows:

11 $$ECE=\widehat{TCE }\times {(I-A)}^{-1}\times Y$$

where *ECE* is the matrix of embodied CO_2_ emissions in provinces. In *ECE*, the *i*th row vector represents the embodied CO_2_ emissions of the financial sector in the *i*th province flows to other provinces; the *i*th column vector represents the embodied CO_2_ emission flow to the *i*th province from other provinces. Thus, the net *ECE* can be calculated as follows:

12 $${NECE}_{i}={ECE}_{i}-{{ECE}_{i}}^{T}$$

where ${NECE}_{i}$ is the net embodied CO_2_ emissions; ${{ECE}_{i}}^{T}$ is the transposed matrix of ${ECE}_{i}$.

### Uncovering the drivers based on kaya identity and LMDI

Based on the net embodied CO_2_ emissions in the interprovincial trade, we use Kaya identity to decompose the drivers that promote net embodied CO_2_ emissions and use LMDI to quantify the driving force, providing a scientific basis for policy-making on low-carbon development in the financial industry. Kaya identity was first proposed by Kaya and Yokobori^61^ and widely expanded to explore the driving factors of greenhouse gas emissions^62,63^. In this study, the embodied CO_2_ emissions of the financial sector can be decomposed as follows:

13 $$NECE=P\times \frac{{GDP}_{s}}{P}\times \frac{{GDP}_{f}}{{GDP}_{s}}\times \frac{NECE}{{GDP}_{f}}=P\times RGPC\times \text{\%}FIN\times NCD$$

where *P* is the population of provinces; *GDP*_*s*_ and *GDP*_*f*_ are the added value of the financial and service sectors; $\frac{GDP}{P}$ represents the per capita added value of the service industry in the provinces (*RGPC*); $\frac{{GDP}_{f}}{{GDP}_{s}}$ represents the percentage of finance in services (*%FIN*); $\frac{NECE}{{GDP}_{f}}$ represents the net CO_2_ density (*NCD*) of financial.

Based on the LMDI method, the Kaya identity can be further decomposed as follows:

14 $$\Delta NECE={NECE}_{t}-{NECE}_{0}$$

where *ΔNECE* is the difference between the *NECE* at *t* year and initial year, in this study, the period is from 2012 to 2020. Then, the logarithmic mean (*W*) can be defined as follows:

15 $$W=\frac{{NECE}_{t}-{NECE}_{0}}{\text{ln}({NECE}_{t}/{NECE}_{0})}$$

Based on the *W*, the *ΔNECE* can be expressed as follows:

16 $$\left\{\begin{array}{c}\Delta P=W\times ln\frac{{P}_{t}}{{P}_{0}}\\ \Delta RGPC=W\times ln\frac{{RGPC}_{t}}{{RGPC}_{0}}\\ \Delta PFIN=W\times ln\frac{{\text{\%}FIN}_{t}}{{\text{\%}FIN}_{0}}\\ \Delta NCD=W\times ln\frac{N{CD}_{t}}{{NCD}_{0}}\end{array}\right.$$

17 $$\Delta NECE=\Delta P+\Delta RGPC+\Delta \text{\%}FIN+\Delta NCD$$

where *ΔP*, *ΔRGPC*, *Δ* $\%FIN$, and* ΔNCD* are the decomposed LMDI variables, which are calculated by the logarithmic mean and logarithm of the difference of these variables at *t* year and initial year (from 2012 to 2015, from 2015 to 2017, from 2017 to 2020, and from 2012 to 2020).

### Data sources

The population and added value were obtained from the National Bureau of Statistics of China in 2013–2021^7^. The CO_2_ emission data of samples in this study were obtained from the China Stock Market Accounting Research Database which followed the “Technical Guide on Carbon Accounting for Financial Institutions (Trial)” (2024) issued by the PBC. Among all listed financial companies, those that had disclosed greenhouse gas emissions data were selected, and individual abnormal data were excluded. The financial data used to calculate the added value of the sample companies were sourced from the Wind database. The MRIO tables of China in 2012, 2015, and 2017 came from the China Emission Accounts & Datasets^64^, which provided complete data on China’s 31 province trade for 42 sectors. It was noted that the MRIO table in 2020 was not yet announced by the China Emission Accounts & Datasets, so we used the linear interpolation combined with the MRIO table from the National Bureau of Statistics of China to establish the matrix of intermediate requirements.

## Results

### CO_2_ emissions of sampled financial enterprises

**Fig. 3:**
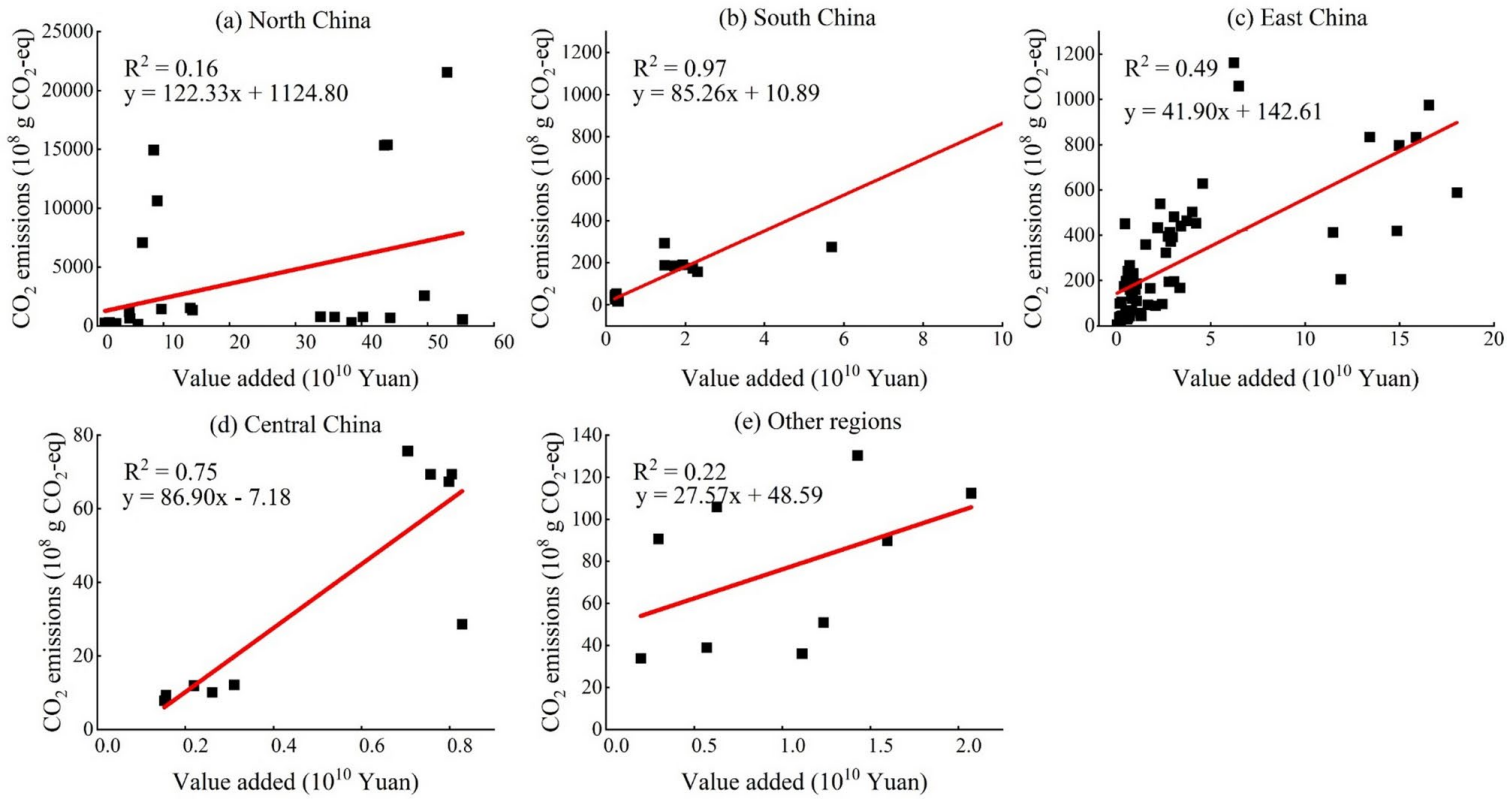
The correlation between enterprise value-added and CO_2_ emissions.

Figure 3 shows the linear fitting results of CO_2_ emissions of sampled enterprises with their value added. South China demonstrates the most favorable fitting effect, with an R^2^ of 0.97. To elaborate, within South China, each 100 yuan increase in value-added yields 85.26 g of CO_2_ emissions. The slope of Fig. 3 (a) is the largest, indicating that the sample companies in North China have the largest value-added carbon intensity, where each 100 yuan increase in value-added yields 122.33 g CO_2_ emissions. Then, the slopes of central and east China are 86.90, 41.90, and 27.57, respectively. Overall, the CO_2_ emissions per added value in the south and central of China are larger than that in the east of China and other regions. The detailed CO_2_ emissions of 134 samples are provided in Table S2.

Based on the linear fitting results of the samples, we found the CO_2_ emissions generated from energy consumption have a significant impact on the total CO_2_ emissions of financial enterprises. The total CO_2_ emissions of over half of financial enterprises were attributed to energy consumption, mainly including electricity usage. More than 90% of the electricity in North China is sourced from coal-fired power generation^65^, leading to the largest slope of North China in Fig. 3 (a). In addition, renewable energy such as hydropower in Sichuan, Yunnan, Guizhou, and Hubei were the main energy resources in southwestern China, leading to the lowest slope of Other regions in Fig. 3 (e)^66^.

### Composition of total CO_2_ emissions from the financial sector by province

**Fig. 4:**
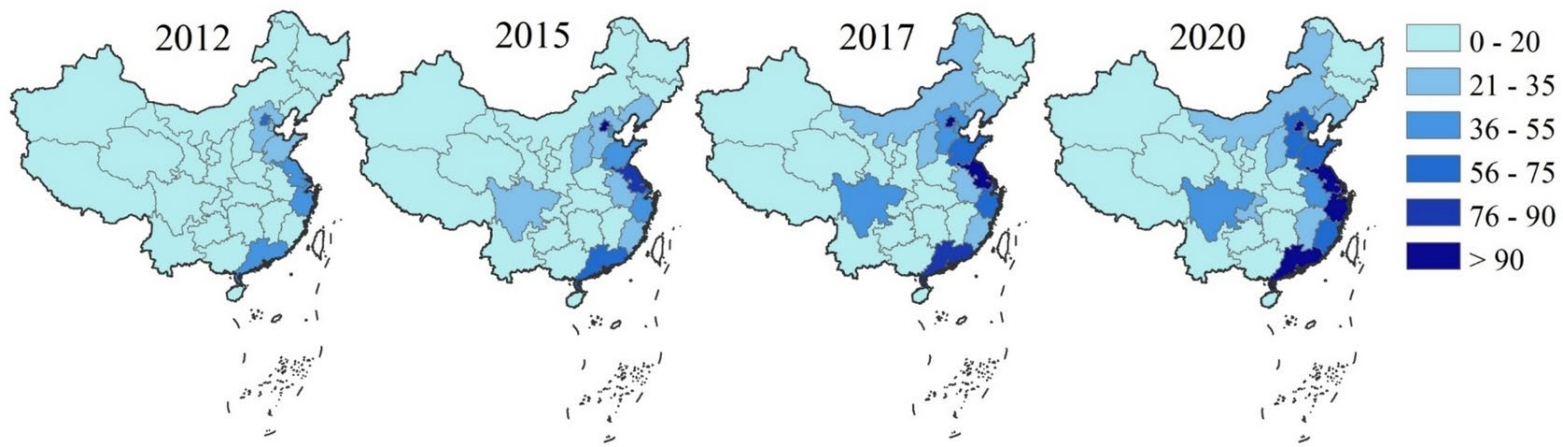
The total CO_2_ emissions of 31 provinces in 2012, 2015, 2017, and 2020 (unit: 10^10^ g CO_2_-eq).

Figure 4 shows the spatial and temporal distribution of the total CO_2_ emissions in the financial sector. The results show the total CO_2_ emissions of the financial sector in China increased from 4.59 to 12.42 Tg CO_2_-eq during 2012-2020, with a 15.28% annual growth rate. Accordingly, the average CO_2_ emissions of 31 provinces increased from 0.15 to 0.40 Tg CO_2_-eq from 2012 to 2020. The largest emitters of CO_2_ were Beijing, Jiangsu, and Shanghai, annual amounting to 1.07, 0.95, and 0.77 Tg CO_2_-eq respectively. In turn, the lowest emitters of CO_2_ were Xizang, Qinghai, and Ningxia, annual amounting to 0.01, 0.02, and 0.03 Tg CO_2_-eq respectively. The detailed results of the total CO_2_ emissions in 31 provinces are provided in Table S3.

During the studied period, the regions with high CO_2_ emissions were concentrated in the southeast coastal areas of China, such as Guangdong, Shanghai, and Jiangsu. In contrast, the regions with relatively low CO_2_ emissions were in the Northwest (Xinjiang, Gansu, and Qinghai) and Southwest China (Yunnan, Guizhou, and Xizang).

### Inflow and outflow CO_2_ emissions of the financial sector via interprovincial trade

**Fig. 5:**
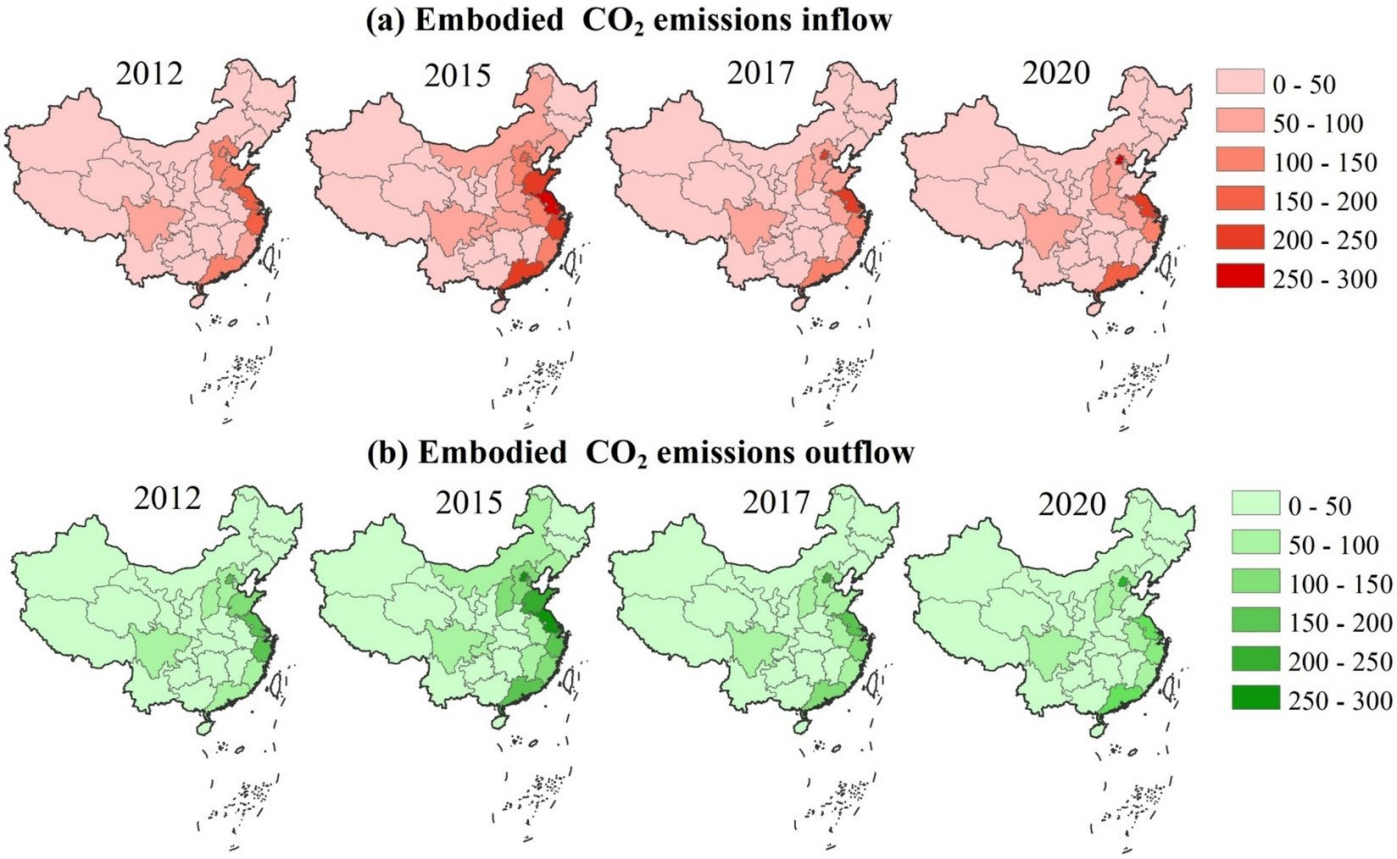
The spatial–temporal pattern of the embodied CO_2_ emissions from interregional trade (unit: 10^10^ g CO_2_-eq).

Figures 5 (a) and (b) show the inflow and outflow of CO_2_ emissions of every province from the financial trade. The provinces with the most inflow of CO_2_ emissions were Jiangsu, Beijing, and Zhejiang, averagely accounting for 2.22, 2.04, and 1.65 Tg CO_2_-eq per year. The provinces with the most outflow CO_2_ emissions are Beijing, Jiangsu, and Shanghai, averagely accounting for 2.44, 2.10, and 1.93 Tg CO_2_-eq per year.

Overall, the transfer of embodied CO_2_ emissions in most provinces exhibited an increasing trend from 2012 to 2015, followed by a subsequent decrease from 2015 to 2020. It is worth noting that the inflow of CO_2_ emissions from Beijing and Shanghai experienced continuous growth throughout the entire study period. Furthermore, both the inflow and outflow of CO_2_ emissions in Xizang displayed a growing trend over the study period. The detailed results of the inflow and outflow of CO_2_ emissions in 31 provinces are provided in Table S4.

### Net CO_2_ emissions of the 31 provinces

**Fig. 6:**
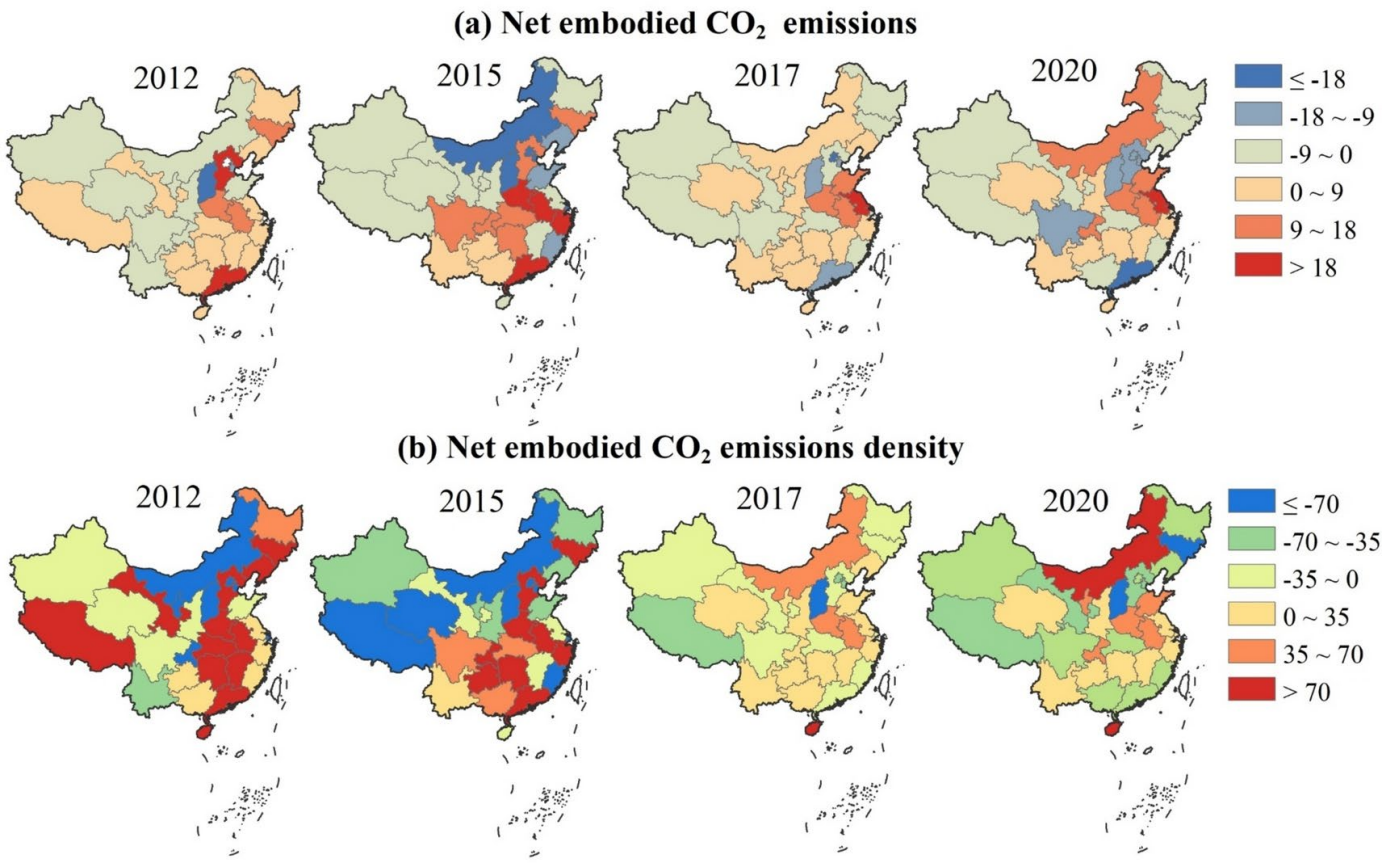
The spatial–temporal pattern of the net embodied CO_2_ emissions and density from interprovincial trade. Unit: (a) 10^10^ g CO_2_-eq; (b) g CO_2_ -eq/10^2^ yuan.

Figure 6 (a) shows the net embodied CO_2_ emissions within the 31 provinces, which is the disparities between inflow and outflow. During the studied period, the provinces with the largest annual net CO_2_ emissions are Anhui, Zhejiang, and Henan, averagely accounting for 0.244, 0.242, and 0.21 Tg CO_2_-eq. The provinces with the lowest annual net CO_2_ emissions are Beijing, Shanghai, and Shanxi, averagely accounting for -0.40, -0.27, and -0.21 Tg CO_2_-eq. It is observed that 5 provinces including Zhejiang, Anhui, Henan, Hunan, and Guizhou, exhibited positive net CO_2_ emissions during the whole studied period. In turn, the 4 provinces exhibit negative net CO_2_ emissions during the whole studied period, including Beijing, Tianjin, Shanxi, and Xinjiang. The detailed results of the net CO_2_ emissions in 31 provinces are provided in Table S5.

Besides, during 2012–2020, 10 provinces, including Fujian, Heilongjiang, Gansu, Xizang, Hubei, Jilin, Hebei, Guangxi, Liaoning, and Guangdong transitioned from net importers to exporters of CO_2_ emissions. Conversely, Nei Mongol, Shaanxi, Shandong, Qinghai, Ningxia, Yunnan, Shanghai, and Chongqing transitioned from net exporters to importers of CO_2_ emissions. Moreover, from 2012 to 2020, we found there was a significant shift in the spatial distribution of net CO_2_ emissions. The regions with high net CO_2_ emissions have transitioned from Central China and its neighbors to East China, Chongqing, and Nei Mongol, while low net CO_2_ emission areas have shifted from North China and the three coastal provinces (Liaoning, Shandong, and Fujian) to Hebei, Sichuan, and Guangdong. Notably, Shanxi consistently maintains a relatively low level of net CO_2_ emissions nationwide. Furthermore, Hebei, Guangdong, and Jilin have transformed from importers of CO_2_ emissions to exporters, whereas Shandong and Jiangsu have reversed this trend, transitioning from exporters to importers. Sichuan, Hubei, and Hunan exhibit a pattern of initial increase followed by a decrease in their net CO_2_ emissions.

Figure 6 (b) shows the *NCD* in the 31 provinces. The provinces with the highest annual *NCD* are Anhui, Jilin, and Henan, amounting to 209.32, 150.18, and 113.09 gCO_2_-eq/10^2^ yuan. The provinces with the lowest annual *NCD* are Shanxi, Tianjin, and Beijing, amounting to -208.12, -118.16, and -112.21 gCO_2_-eq/10^2^ yaun. The absolute values of the *NCD* in Gansu (0.45 gCO_2_-eq/10^2^ yaun), Sichuan (1.34), and Yunnan (4.06) are minimal, thereby indicating a relative equilibrium in the import and export of CO_2_ emissions in these provinces.

The *NCD* of 16 provinces was reduced from 2012 to 2020. Therein, the *NCD* of Hebei and Jilin showed the most significant reductions, with a reduction of 134.31% and 117.55%. The results indicate that provinces with a relatively high CO_2_ emission density at the commencement of the study period underwent a more considerable decline in their *NCD*.

### Transfer of embodied CO_2_ emissions of 31 provinces

**Fig. 7:**
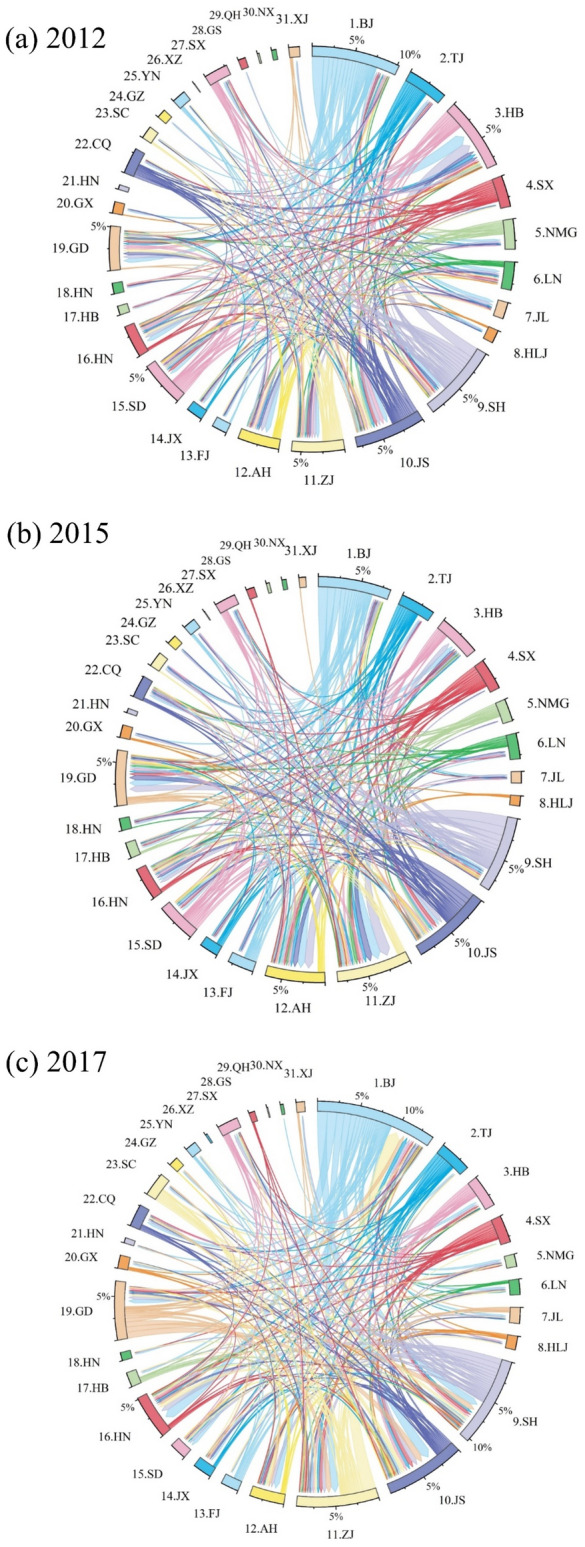
The transfer networks of financial embodied CO_2_ emissions in interprovincial trade (unit: 10^6^ g CO_2_-eq).

Figure 7 shows the transfer networks of financial embodied CO_2_ emissions of 31 provinces in interprovincial trade, and the CO_2_ emissions transferred within the local province had been excluded. In 2012, Beijing was the largest exporter of CO_2_ emissions, and primarily exported to Hebei, Anhui, and Henan, accounting for 17.56%, 8.11%, and 6.76% in its outflow emissions (0.60 Tg CO_2_-eq), respectively. Following, Shanghai exported 19.82%, 9.10%, and 7.08% of its emissions outflow (0.47 Tg CO_2_-eq) to Hebei, Anhui, and Henan. Jiangsu was the third largest exporter (0.27 Tg CO_2_-eq) of CO_2_ emissions, mainly exporting to Hebei (11.41%), Guangdong (11.02%), and Zhejiang (6.93%). In 2015, Shanghai, Beijing, and Jiangsu continued to be the largest exporters of CO_2_ emissions (0.554, 0.546, and 0.386 Tg CO_2_-eq), and their largest export destinations were Anhui, Zhejiang, and Guangdong. In 2017 and 2020, Beijing and Shanghai were the top two emitters of CO_2_ outflows, primarily directed to Jiangsu, Zhejiang, and Henan. Beijing and Shanghai exported the most to Jiangsu, accounting for 13.03% and 10.97% of their respective total export.

Overall, Beijing is the largest exporter of CO_2_ emissions among 31 provinces with an average of 0.73 Tg CO_2_-eq per year, mainly exporting to Zhejiang (10.51%), Hebei (9.50%), and Anhui (9.59%) during 2012–2020. Jiangsu is the largest importer with an average of 0.47 Tg CO_2_-eq per year, mainly importing from Beijing (13.35%), Shanghai (9.90%), and Shanxi (7.91%). Anhui, Zhejiang, and Henan contributed the largest net CO_2_ emissions during 2012–2020, accounting for 0.98, 0.97, and 0.84 Tg CO_2_-eq, respectively. Besides, Beijing, Shanghai, and Shanxi had the lowest total net CO_2_ emissions during the studied period, accounting for -1.62, -1.09, and -0.83 Tg CO_2_-eq, respectively. The inflows and outflows of 7 provinces (including Xinjiang, Xizang, Qinghai, Hainan, Ningxia, Gansu, and Guizhou) are lower than 1% of the total inflow and outflow of CO_2_ emissions, indicating a little connection of the trade with other provinces. Further, the inflows and outflows of 4 provinces (including Ningxia, Yunnan, Qinghai, and Xizang) are lower than 1% of the maximum absolute value, which means a balanced situation of CO_2_ emissions among these 4 provinces.

### Driving factors of embodied CO_2_ emissions

**Fig. 8:**
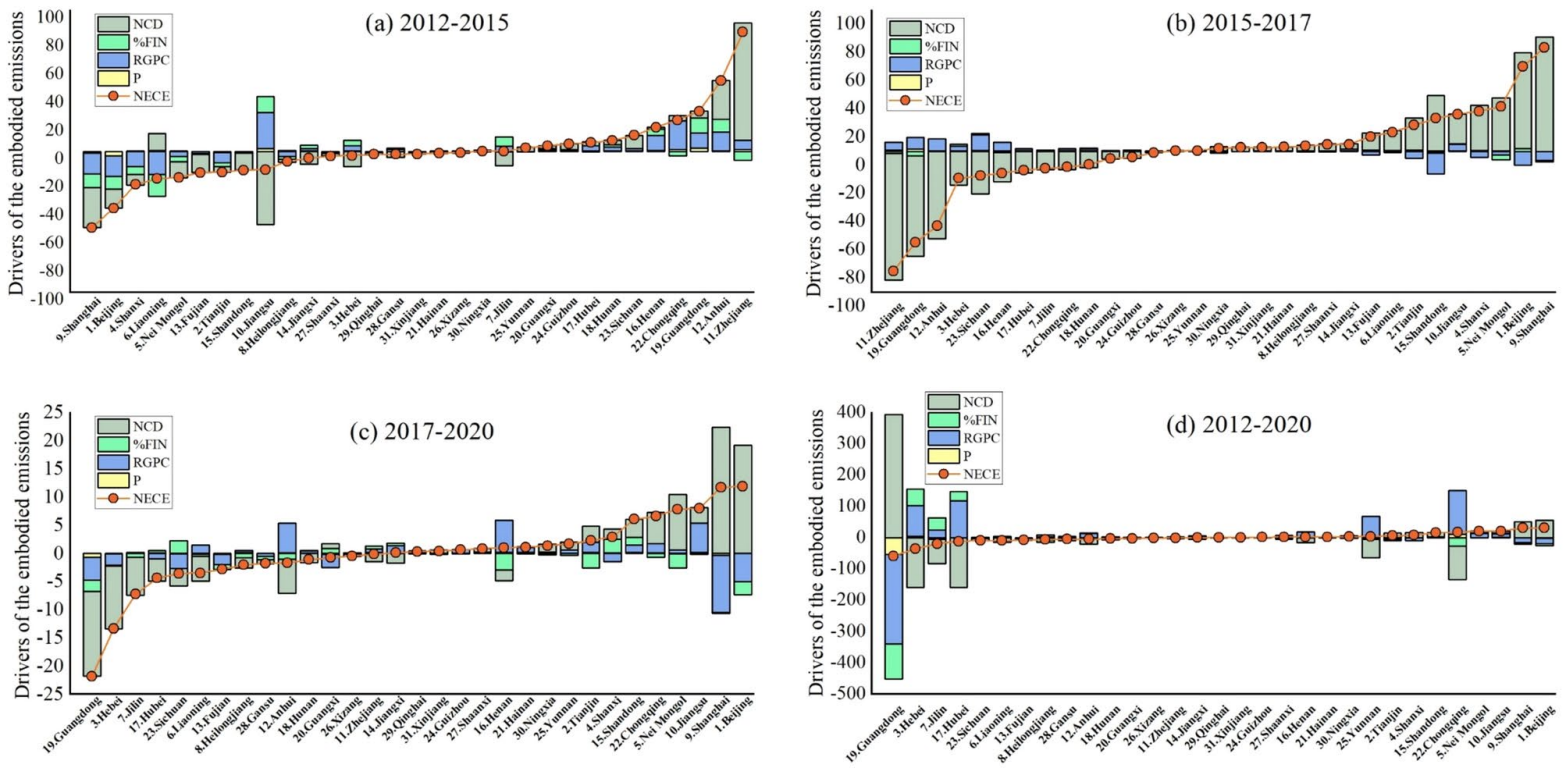
Driving factor contribution of the embodied CO_2_ emissions (unit: 10^10^ g CO_2_-eq).

As Fig. 8 shows, the increase in embodied CO_2_ emissions was decomposed into four factors by the Kaya identity: population (*P*), per capita value added in the service sector (*RGPC*), share of financial in service (*%FIN*), and net CO_2_ emissions density (*NCD*). During the whole studied period, the contribution of *NCD*, *RGPC*, *%FIN*, and *P* were 47.08%, 37.54%, 12.32%, and 3.06% respectively. During 2012–2015, *NCD* was the primary contributor to the increase or decrease of embodied CO_2_ emissions, accounting for 47.83% or 52.53%. In 2015–2017, the average contribution of *NCD* increased to 82.96%. The following contributor was *RGPC*, with an average proportion of 12.90%. While the contributions of *P* and *%FIN* were both less than 5%. In 2017–2020, the average contribution of *NCD* decreased to 52.66%, while the average contribution of *RGPC* increased to 30.04%.

During the studied period, the *NCD* of Shaanxi, Jiangxi, Xizang, and Guangdong, exhibited a negative correlation with the net CO_2_ emissions. For example, the increase in *NCD* was lower than the decrease in *P*, *%FIN*, and *RGPC* in Xizang and Guangdong, leading to a negative relationship between *NCD* and net CO_2_ emissions. In contrast, the decrease in *NCD* was lower than the increase of *P*, *RGPC*, and *%FIN* in Jiangxi and Shaanxi, leading to an increase in net CO_2_ emissions. In addition, the *RGPC* of Beijing, Shanghai, and Zhejiang showed a negative relationship with the net CO_2_ emissions. These provinces had relatively high economic development and technological advance levels, thereby leading to a decoupling between financial industry development and CO_2_ emissions. While the 13 provinces in northwest and east China did not show such decoupling.

In conclusion, the driving factors of net CO_2_ emissions in the financial sector ranked in importance as *NCD* > *RGPC* > *%FIN* > *P* during the whole studied period. The trend of *NCD* contributing to the net CO_2_ emissions increased in 2012–2015 and then decreased in 2015–2020. While the trend in the contribution of *RGPC* was the opposite. The average contribution of *P* of 31 provinces was minimal during the studied period. However, the contribution of *P* is significant in Jiangxi and Jilin, accounting for 9.43% and 10.54%, respectively. Besides, *RGPC* is the primary factor influencing net CO_2_ emissions in Ningxia, with an average contribution of 71.99%.

## Discussion

During the studied period, the total CO_2_ emissions of the financial sector increased significantly at an annual average growth rate of 15.28%, peaking between 2012 and 2015. The CO_2_ emissions from the financial sector mainly originate from East China where the financial industries are relatively well-developed. The central and western regions have comparatively lower CO_2_ emissions. This spatial pattern aligns with the spatial structure of China’s financial industry, which suggests that the growth of the financial industry’s value-added was not decoupled from its CO_2_ emissions during the studied period. For example, Sichuan, Shanxi, and Nei Mongol, which were influenced by the optimization and upgrading of industrial structure and economic restructuring, showed a significant increase of value added in the financial sector during the research period, with a growth of 192.46%, 135.90%, and 116.96%, respectively. Consequently, these provinces transformed from low CO_2_ emissions to major sources of emissions.

The inflow and outflow of CO_2_ emissions have a positive relationship with the level of trade development. The provinces with rapid development of the provincial trade had a relatively large amount of inflow and outflow CO_2_ emissions, including Beijing, Jiangsu, and Zhejiang. In contrast, provinces of central and western China, such as Hainan, Qinghai, and Xizang, with less provincial trade, had a relatively lower inflow and outflow of CO_2_ emissions. The net CO_2_ emissions of the 14 provinces decreased in the studied period. Provinces like Shandong, Qinghai, and Hainan have very limited involvement in provincial trade, therefore, CO_2_ emissions of the financial sector in these provinces mainly come from local sources, resulting in a low amount of the net CO_2_ emissions.

Provinces with relatively developed financial sectors such as Shanghai, Beijing, and Tianjin, offer extensive financial services to other provinces. Thus, the local CO_2_ emissions of these provinces outflow to other provinces, leading to negative net CO_2_ emissions. Provinces like Henan and Shandong, rely heavily on resources yet exhibit underdeveloped financial sectors, resulting in substantial positive net CO_2_ emissions. Besides, a large amount of CO_2_ emissions transfer among the neighboring provinces. For example, the provinces such as Zhejiang, Henan, and Anhui, import a large amount of transferring CO_2_ emissions from their neighbors, Shanghai and Jiangsu. Similarly, Guangdong exports a large amount of CO_2_ emissions to its neighbors, Guangxi, Guizhou, and Hunan.

## Conclusions and implications

The study provides a framework for estimating the total CO_2_ emissions of the financial sector at a provincial scale. It also assesses the embodied CO_2_ emissions in interprovincial financial trade and identifies the drivers behind these emissions. This aims to provide a foundation for reducing CO_2_ emissions and formulating carbon tax policies within China’s financial sector. The conclusions are shown as follows:

First, the total CO_2_ emissions of the financial sector in China increased from 4.59 to 12.42 Tg CO_2_-eq from 2012 to 2020, with an annual growth rate of 15.28%. The provinces with large-scale financial industries, such as Beijing, Shanghai, Jiangsu, and Guangdong, emerged as the primary contributors to CO_2_ emissions. Second, Beijing was the largest exporter of CO_2_ emissions among 31 provinces, releasing 2.44 Tg CO_2_-eq annually and mainly exporting to Zhejiang (10.51%), Anhui (9.59%), and Hebei (9.50%). Conversely, Jiangsu was the largest importer with an average of 2.22 Tg CO_2_-eq per year, mainly importing from Beijing (13.35%), Shanghai (9.90%), and Shanxi (7.91%). Anhui emerged as the largest net CO_2_ emitter, accounting for an average of 0.244 Tg CO_2_-eq annually, exhibiting the highest annual net CO_2_ intensity at 2.09 gCO_2_-eq per yuan. Third, during the whole studied period, the drivers of CO_2_ emissions within the financial sector were ranked in order of importance as follows: *NCD* > *RGPC* > *%FIN* > *P*. Therein, the contribution of *NCD* exhibited an initial increase followed by a decrease over the studied periods, averagely constituting 47.08% of the net CO_2_ emissions. Provinces with developed financial sectors exhibited a decoupling between financial industry growth and CO_2_ emissions.

Based on the above conclusions, this study provides policymakers as follows: first, we suggested the provinces that are the concentration of China’s financial sector’s major sources of CO_2_ emissions (such as Guangdong, Shanghai, and Jiangsu) give priority to reducing the CO_2_ emission intensity of the financial industry. For example, they can transfer parts of their financial industry to the regions with less developed financial sectors, such as Qinghai, Guizhou, and Sichuan, which are in the northwest and northeast of China. Second, we found that the rapid growth of the financial industry within the trade across provinces complicates the tracking of embodied CO_2_ emissions and leads to large carbon leakages. A more equal reduction responsibility of CO_2_ emissions between producers and consumers is needed, therefore, policymakers can utilize the framework provided in this study to format detailed carbon tax policies for each province according to their import, export, and net CO_2_ emissions. Third, we found that the *NCD* is the main driver of the net CO_2_ emissions in the majority of provinces, hence the *NCD* reduction should be given higher priority than the other drivers. Green technology progress (GTP) is considered an effective path for improving carbon efficiency^67^, which requires the assistance of relevant environmental regulations^68^. Furthermore, the innovation environment may influence the CO_2_ emission reduction effect of GTP^69^. For example, improving the green electricity rate used by financial enterprises, and implementing paperless office strategies between their branch offices. Fourth, we found variations in their driving factors among the 31 provinces and solely addressing the singular factor accountable for the net CO_2_ emissions may not effectively reduce the emissions. For example, *RGPC* and *%FIN* are both significant drivers in Ningxia during the study period, therefore, reducing net CO_2_ emissions requires controlling both *RGPC* and *%FIN*. Moreover, for Jiangxi and Jilin, it is effective in reducing the net CO_2_ emissions by controlling both the *NCD* and *P*. Finally, policymakers should prioritize the use of relative indicators (such as *NCD*) rather than absolute CO_2_ reductions when setting goals for the financial sectors. Simultaneously, financial sectors in each province should collaborate with environmental, energy, trade, transport, and other relevant sectors to devise tailored green financial development policies.

However, several limitations persist in this study. Firstly, the consideration of embodied emissions was limited to the years 2012, 2015, 2017, and 2020, primarily due to the unavailability of annual MRIO tables focusing on China. Additionally, the absence of the intermediate requirements matrix for 2020 poses challenges in tracing transferred CO_2_ emissions within the trade. While the use of interpolated data in 2020 brings potential uncertainty, this issue can be promptly addressed in future research once the MRIO tables become accessible. Then the obtained financial enterprises included in the study, comprising 134 samples from the China Stock Market & Accounting Research Database, represent over 90% of the market capitalization of listed financial enterprises, yet emissions from unlisted financial sectors like private banks, private fund providers, private financial institutions, and pawnshops were not accounted for. Subsequent studies could enhance precision by collecting data through detailed site investigations on these unlisted financial enterprises. Overall, this study provides a foundation for future related studies by establishing a framework to trace the embodied CO_2_ emissions of the financial sector.

## Supplementary Information

Supplementary Information 1.

Supplementary Information 2.

## Data Availability

The datasets used and/or analyzed during the current study are available from the corresponding author upon reasonable request.
