# Supplementary material for: Tracing embodied CO2 emissions and drivers in China’s financial industry under inter-provincial trade
Source: Sci Rep. 2024 Nov 19;14:28668. doi: 10.1038/s41598-024-79833-x (PMC11577053; doi:10.1038/s41598-024-79833-x)
Supplement: Supplementary file 1 — Supplementary Information 1. [file 41598_2024_79833_MOESM1_ESM.docx]

| **Nomenclature** | | | |
| --- | --- | --- | --- |
| CO_2_ | Carbon dioxide | TCE | Total CO_2_ emissions |
| MRIO | Multi-regional input-output model | P | Population |
| GDP | Gross domestic product | RGPC | Per capita added value of the service industry |
| %FIN | The proportion of the value added by the financial sector in the service sector | NCD | The density of net embodied CO_2_ emissions in the financial sector |
