# Supplementary material for: Tracing embodied CO2 emissions and drivers in China’s financial industry under inter-provincial trade
Source: Sci Rep. 2024 Nov 19;14:28668. doi: 10.1038/s41598-024-79833-x (PMC11577053; doi:10.1038/s41598-024-79833-x)
Supplement: Supplementary file 2 — Supplementary Information 2. [file 41598_2024_79833_MOESM2_ESM.docx]

**Supplementary Information for**

### Tracing embodied CO_2_ emissions and drivers in China's financial industry under inter-provincial trade

**Table S1 The value added of the financial industry (unit: 10^8^ yuan).**

| Provinces | Region | 2012 | 2015 | 2017 | 2020 |
| --- | --- | --- | --- | --- | --- |
| 1.Beijing | North China | 2536.91 | 3926.28 | 4655.37 | 7187.97 |
| 2.Tianjin | North China | 1001.59 | 1603.23 | 1951.75 | 2056.73 |
| 3.Hebei | North China | 913.66 | 1480.92 | 2053.44 | 2599.55 |
| 4.Shanxi | North China | 639.61 | 1140.54 | 1320.05 | 1207.68 |
| 5.Nei Mongol | North China | 502.01 | 829.20 | 1099.85 | 888.90 |
| 6.Liaoning | Other Regions | 969.37 | 1869.46 | 1964.58 | 2103.11 |
| 7.Jilin | Other Regions | 244.63 | 565.27 | 709.64 | 901.24 |
| 8.Heilongjiang | Other Regions | 485.11 | 847.66 | 932.35 | 1052.94 |
| 9.Shanghai | East China | 2450.36 | 4162.70 | 5330.54 | 7166.26 |
| 10.Jiangsu | East China | 3136.51 | 5302.93 | 6783.87 | 8405.79 |
| 11.Zhejiang | East China | 2762.24 | 2922.93 | 3533.05 | 5590.60 |
| 12.Anhui | East China | 617.62 | 1241.87 | 1663.59 | 2553.94 |
| 13.Fujian | East China | 1015.37 | 1681.33 | 2055.53 | 3418.36 |
| 14.Jiangxi | East China | 413.07 | 897.65 | 1107.12 | 1808.63 |
| 15.Shandong | East China | 1936.11 | 2994.66 | 3651.56 | 4567.36 |
| 16.Henan | Central China | 1013.60 | 1991.11 | 2509.19 | 2955.87 |
| 17.Hubei | Central China | 870.36 | 1853.12 | 2640.86 | 3027.37 |
| 18.Hunan | Central China | 579.76 | 1104.18 | 1610.31 | 2126.44 |
| 19.Guangdong | South China | 3171.96 | 5757.08 | 6853.01 | 9906.99 |
| 20.Guangxi | South China | 573.05 | 1018.01 | 1273.40 | 1598.00 |
| 21.Hainan | South China | 130.69 | 242.82 | 308.94 | 397.91 |
| 22.Chongqing | Other Regions | 915.65 | 1410.18 | 1813.73 | 2212.80 |
| 23.Sichuan | Other Regions | 1303.56 | 2202.23 | 3203.27 | 3375.75 |
| 24.Guizhou | Other Regions | 365.87 | 607.11 | 787.88 | 1141.71 |
| 25.Yunnan | Other Regions | 541.18 | 981.85 | 1194.67 | 1500.41 |
| 26.Xizang | Other Regions | 32.04 | 68.05 | 110.20 | 138.56 |
| 27.Shaanxi | Other Regions | 551.20 | 1082.37 | 1300.10 | 1820.90 |
| 28.Gansu | Other Regions | 184.43 | 443.12 | 553.59 | 897.52 |
| 29.Qinghai | Other Regions | 83.73 | 220.87 | 274.60 | 263.67 |
| 30.Ningxia | Other Regions | 167.48 | 256.38 | 314.69 | 321.07 |
| 31.Xinjiang | Other Regions | 360.40 | 563.80 | 623.53 | 1086.45 |

**Table S2 The sample data of listed companies of the financial sector.**

**(**CE: CO_2_ emissions; CPE: Cash paid for employees; NAP: Net profit attributable to shareholders of the parent company; VTF: Various taxes and fees paid; DA: Depreciation of assets; AIA: Amortization of intangible assets; VA: Value added; VAF: Value-added emission factor**)**

| Enterprise name | Year | Stock code | Region | CE(10^6^ g CO_2_-eq) | CPE (yuan) | NAP  (yuan) | VTF (yuan) | DA (yuan) | AIA  (yuan) | VA  (yuan) | VAF  (g CO2-eq/yuan) |
| --- | --- | --- | --- | --- | --- | --- | --- | --- | --- | --- | --- |
| Guoyuan Securities Company Limited | 2020 | 000728.SZ | East China | 3.97E+03 | 1.43E+09 | 1.37E+09 | 6.42E+08 | 7.89E+07 | 3.22E+07 | 3.55E+09 | 1.12 |
| Guoyuan Securities Company Limited | 2021 | 000728.SZ | East China | 4.51E+04 | 1.70E+09 | 1.91E+09 | 7.27E+08 | 8.02E+07 | 3.58E+07 | 4.46E+09 | 10.12 |
| Holly Futures Co., Ltd. | 2022 | 001236.SZ | East China | 5.88E+02 | 1.78E+08 | 1.24E+07 | 6.28E+07 | 5.85E+06 | 5.35E+05 | 2.60E+08 | 2.26 |
| Bank of Qingdao Co.,Ltd. | 2019 | 002948.SZ | East China | 4.39E+03 | 1.62E+09 | 2.28E+09 | 1.27E+09 | 4.10E+08 | 0.00E+00 | 5.59E+09 | 0.79 |
| Bank of Qingdao Co.,Ltd. | 2020 | 002948.SZ | East China | 3.70E+03 | 1.68E+09 | 2.39E+09 | 1.60E+09 | 4.29E+08 | 0.00E+00 | 6.10E+09 | 0.61 |
| Bank of Qingdao Co.,Ltd. | 2021 | 002948.SZ | East China | 4.39E+03 | 1.93E+09 | 2.92E+09 | 1.80E+09 | 4.93E+08 | 0.00E+00 | 7.15E+09 | 0.61 |
| Bank of Suzhou Co.,Ltd | 2022 | 002966.SZ | East China | 2.32E+04 | 2.33E+09 | 3.92E+09 | 2.18E+09 | 2.17E+08 | 1.01E+08 | 8.75E+09 | 2.65 |
| East Money Information Co., Ltd. | 2023 | 300059.SZ | East China | 5.64E+03 | 2.40E+09 | 8.19E+09 | 1.87E+09 | 3.36E+08 | 3.24E+07 | 1.28E+10 | 0.44 |
| Shanghai Pudong Development Bank Co.,Ltd. | 2021 | 600000.SH | East China | 4.13E+04 | 2.76E+10 | 5.30E+10 | 2.68E+10 | 7.26E+09 | 0.00E+00 | 1.15E+11 | 0.36 |
| Shanghai Pudong Development Bank Co.,Ltd. | 2022 | 600000.SH | East China | 2.06E+04 | 3.13E+10 | 5.12E+10 | 2.86E+10 | 7.83E+09 | 0.00E+00 | 1.19E+11 | 0.17 |
| SDIC Capital Co., Ltd | 2022 | 600061.SH | East China | 1.86E+04 | 4.51E+09 | 2.94E+09 | 1.78E+09 | 1.74E+08 | 1.39E+08 | 9.55E+09 | 1.95 |
| Zhejiang Orient Financial Holdings Group Co.,Ltd. | 2022 | 600120.SH | East China | 2.41E+02 | 5.92E+08 | 9.47E+08 | 4.89E+08 | 4.91E+07 | 4.36E+07 | 2.12E+09 | 0.11 |
| State Grid Yingda Co.,Ltd. | 2022 | 600517.SH | East China | 1.74E+04 | 9.05E+08 | 1.07E+09 | 1.94E+09 | 1.28E+08 | 5.50E+07 | 4.10E+09 | 4.25 |
| Haitong Securities Co., Ltd. | 2018 | 600837.SH | East China | 9.26E+03 | 6.65E+09 | 5.21E+09 | 4.32E+09 | 3.01E+08 | 1.92E+08 | 1.67E+10 | 0.56 |
| Haitong Securities Co., Ltd. | 2019 | 600837.SH | East China | 8.96E+03 | 6.21E+09 | 9.52E+09 | 4.40E+09 | 4.18E+08 | 1.91E+08 | 2.07E+10 | 0.43 |
| Haitong Securities Co., Ltd. | 2020 | 600837.SH | East China | 9.72E+03 | 6.87E+09 | 1.09E+10 | 5.72E+09 | 5.49E+08 | 2.06E+08 | 2.42E+10 | 0.40 |
| Haitong Securities Co., Ltd. | 2021 | 600837.SH | East China | 4.14E+04 | 7.97E+09 | 1.28E+10 | 6.57E+09 | 6.93E+08 | 2.12E+08 | 2.83E+10 | 1.46 |
| Haitong Securities Co., Ltd. | 2022 | 600837.SH | East China | 5.40E+04 | 8.95E+09 | 6.55E+09 | 6.63E+09 | 7.93E+08 | 2.11E+08 | 2.31E+10 | 2.33 |
| Huaan Securities Co.,Ltd. | 2021 | 600909.SH | East China | 4.43E+03 | 1.05E+09 | 1.42E+09 | 7.02E+08 | 5.33E+07 | 3.44E+07 | 3.26E+09 | 1.36 |
| Orient Securities Company Limited | 2016 | 600958.SH | East China | 1.34E+04 | 2.97E+09 | 2.31E+09 | 2.54E+09 | 7.58E+07 | 4.78E+07 | 7.95E+09 | 1.69 |
| Orient Securities Company Limited | 2017 | 600958.SH | East China | 1.61E+04 | 3.12E+09 | 3.55E+09 | 4.55E+08 | 8.49E+07 | 5.80E+07 | 7.28E+09 | 2.21 |
| Orient Securities Company Limited | 2018 | 600958.SH | East China | 2.92E+03 | 3.43E+09 | 1.23E+09 | 8.40E+08 | 1.07E+08 | 6.31E+07 | 5.67E+09 | 0.51 |
| Orient Securities Company Limited | 2019 | 600958.SH | East China | 5.53E+03 | 3.30E+09 | 2.44E+09 | 6.72E+08 | 1.70E+08 | 7.09E+07 | 6.65E+09 | 0.83 |
| Orient Securities Company Limited | 2022 | 600958.SH | East China | 1.60E+04 | 4.94E+09 | 3.01E+09 | 1.25E+09 | 5.85E+08 | 1.34E+08 | 9.92E+09 | 1.61 |
| Bank of Nanjing Co.,Ltd. | 2021 | 601009.SH | East China | 4.81E+04 | 7.05E+09 | 1.59E+10 | 6.79E+09 | 5.94E+08 | 1.43E+08 | 3.04E+10 | 1.58 |
| Bank of Nanjing Co.,Ltd. | 2022 | 601009.SH | East China | 4.42E+04 | 7.84E+09 | 1.84E+10 | 7.05E+09 | 6.13E+08 | 1.72E+08 | 3.41E+10 | 1.30 |
| Caitong Securities Co.,Ltd | 2021 | 601108.SH | East China | 3.80E+03 | 1.97E+09 | 2.57E+09 | 1.03E+09 | 1.02E+08 | 7.09E+07 | 5.75E+09 | 0.66 |
| Caitong Securities Co.,Ltd | 2022 | 601108.SH | East China | 3.71E+03 | 2.27E+09 | 1.52E+09 | 9.42E+08 | 9.18E+07 | 7.82E+07 | 4.90E+09 | 0.76 |
| Jiangsu Changshu Rural Commercial Bank Co.,Ltd. | 2021 | 601128.SH | East China | 1.98E+04 | 1.97E+09 | 2.19E+09 | 7.95E+08 | 1.29E+08 | 2.42E+07 | 5.10E+09 | 3.87 |
| Jiangsu Changshu Rural Commercial Bank Co.,Ltd. | 2022 | 601128.SH | East China | 2.43E+04 | 2.12E+09 | 2.74E+09 | 1.01E+09 | 1.45E+08 | 4.39E+07 | 6.06E+09 | 4.01 |
| Jiangsu Changshu Rural Commercial Bank Co.,Ltd. | 2023 | 601128.SH | East China | 2.67E+04 | 2.24E+09 | 3.28E+09 | 1.26E+09 | 1.55E+08 | 5.51E+07 | 6.98E+09 | 3.83 |
| Industrial Bank Co.,Ltd. | 2021 | 601166.SH | East China | 4.20E+04 | 3.01E+10 | 8.27E+10 | 3.33E+10 | 2.25E+09 | 2.18E+08 | 1.49E+11 | 0.28 |
| Guotai Junan Securities Co., Ltd. | 2021 | 601211.SH | East China | 3.92E+04 | 8.73E+09 | 1.50E+10 | 5.46E+09 | 3.62E+08 | 1.99E+08 | 2.98E+10 | 1.32 |
| Guotai Junan Securities Co., Ltd. | 2022 | 601211.SH | East China | 3.96E+04 | 9.69E+09 | 1.15E+10 | 5.47E+09 | 3.92E+08 | 2.25E+08 | 2.73E+10 | 1.45 |
| Bank of Shanghai Co., Ltd. | 2022 | 601229.SH | East China | 5.03E+04 | 6.69E+09 | 2.23E+10 | 9.54E+09 | 1.70E+09 | 0.00E+00 | 4.02E+10 | 1.25 |
| Bank of Communications Co.,Ltd. | 2017 | 601328.SH | East China | 8.33E+04 | 2.70E+10 | 7.02E+10 | 2.77E+10 | 9.32E+09 | 0.00E+00 | 1.34E+11 | 0.62 |
| Bank of Communications Co.,Ltd. | 2019 | 601328.SH | East China | 7.96E+04 | 3.11E+10 | 7.73E+10 | 2.73E+10 | 1.40E+10 | 0.00E+00 | 1.50E+11 | 0.53 |
| Bank of Communications Co.,Ltd. | 2020 | 601328.SH | East China | 8.32E+04 | 3.21E+10 | 7.83E+10 | 3.35E+10 | 1.48E+10 | 0.00E+00 | 1.59E+11 | 0.52 |
| Bank of Communications Co.,Ltd. | 2021 | 601328.SH | East China | 9.75E+04 | 3.39E+10 | 8.76E+10 | 2.92E+10 | 1.48E+10 | 0.00E+00 | 1.66E+11 | 0.59 |
| Bank of Communications Co.,Ltd. | 2022 | 601328.SH | East China | 5.88E+04 | 3.70E+10 | 9.21E+10 | 3.51E+10 | 1.62E+10 | 0.00E+00 | 1.80E+11 | 0.33 |
| Industrial Securities Co.,Ltd. | 2018 | 601377.SH | East China | 5.07E+03 | 3.43E+09 | 1.35E+08 | 2.11E+09 | 8.14E+07 | 5.06E+07 | 5.81E+09 | 0.87 |
| Industrial Securities Co.,Ltd. | 2019 | 601377.SH | East China | 6.84E+03 | 3.35E+09 | 1.76E+09 | 2.87E+09 | 8.93E+07 | 7.11E+07 | 8.14E+09 | 0.84 |
| Industrial Securities Co.,Ltd. | 2020 | 601377.SH | East China | 4.46E+03 | 4.05E+09 | 4.00E+09 | 4.89E+09 | 9.62E+07 | 1.13E+08 | 1.32E+10 | 0.34 |
| Industrial Securities Co.,Ltd. | 2021 | 601377.SH | East China | 3.60E+04 | 5.06E+09 | 4.74E+09 | 5.38E+09 | 1.18E+08 | 1.68E+08 | 1.55E+10 | 2.33 |
| Guolian Securities Co., Ltd. | 2020 | 601456.SH | East China | 3.83E+03 | 6.21E+08 | 5.88E+08 | 2.50E+08 | 2.36E+07 | 4.39E+07 | 1.53E+09 | 2.51 |
| Guolian Securities Co., Ltd. | 2021 | 601456.SH | East China | 4.26E+03 | 1.02E+09 | 8.89E+08 | 3.63E+08 | 2.88E+07 | 5.13E+07 | 2.35E+09 | 1.81 |
| Guolian Securities Co., Ltd. | 2022 | 601456.SH | East China | 3.18E+03 | 1.27E+09 | 7.67E+08 | 3.23E+08 | 3.32E+07 | 5.63E+07 | 2.45E+09 | 1.30 |
| Zhejiang Shaoxing Ruifeng Rural Commercial Bank Co.,Ltd | 2022 | 601528.SH | East China | 1.06E+04 | 7.68E+08 | 1.53E+09 | 2.94E+08 | 8.45E+07 | 4.57E+06 | 2.68E+09 | 3.94 |
| Soochow Securities Co.,Ltd. | 2021 | 601555.SH | East China | 6.50E+03 | 2.06E+09 | 2.39E+09 | 1.20E+09 | 8.63E+07 | 5.36E+07 | 5.79E+09 | 1.12 |
| Soochow Securities Co.,Ltd. | 2022 | 601555.SH | East China | 6.15E+03 | 2.31E+09 | 1.74E+09 | 6.32E+08 | 9.91E+07 | 5.70E+07 | 4.84E+09 | 1.27 |
| China Pacific Insurance (Group) Co., Ltd. | 2016 | 601601.SH | East China | 4.53E+04 | 1.64E+10 | 1.21E+10 | 1.22E+10 | 1.20E+09 | 4.22E+08 | 4.22E+10 | 1.07 |
| China Pacific Insurance (Group) Co., Ltd. | 2017 | 601601.SH | East China | 6.29E+04 | 1.88E+10 | 1.47E+10 | 1.03E+10 | 1.42E+09 | 4.68E+08 | 4.57E+10 | 1.38 |
| China Pacific Insurance (Group) Co., Ltd. | 2020 | 601601.SH | East China | 1.16E+05 | 2.33E+10 | 2.46E+10 | 1.18E+10 | 1.76E+09 | 7.50E+08 | 6.22E+10 | 1.87 |
| China Pacific Insurance (Group) Co., Ltd. | 2021 | 601601.SH | East China | 1.06E+05 | 2.54E+10 | 2.68E+10 | 9.85E+09 | 1.78E+09 | 8.66E+08 | 6.48E+10 | 1.64 |
| Huatai Securities Co.,Ltd. | 2017 | 601688.SH | East China | 1.66E+04 | 5.59E+09 | 9.28E+09 | 2.50E+09 | 2.44E+08 | 2.83E+08 | 1.79E+10 | 0.93 |
| Huatai Securities Co.,Ltd. | 2021 | 601688.SH | East China | 3.73E+04 | 9.34E+09 | 1.33E+10 | 5.24E+09 | 4.04E+08 | 4.77E+08 | 2.88E+10 | 1.30 |
| Huatai Securities Co.,Ltd. | 2022 | 601688.SH | East China | 3.24E+04 | 1.07E+10 | 1.11E+10 | 3.53E+09 | 4.70E+08 | 4.30E+08 | 2.62E+10 | 1.23 |
| Everbright Securities Company Limited | 2017 | 601788.SH | East China | 1.21E+04 | 3.44E+09 | 3.02E+09 | 1.68E+09 | 1.29E+08 | 2.30E+08 | 8.50E+09 | 1.43 |
| Everbright Securities Company Limited | 2018 | 601788.SH | East China | 1.78E+04 | 3.58E+09 | 1.03E+08 | 1.52E+09 | 1.23E+08 | 2.08E+08 | 5.54E+09 | 3.21 |
| Everbright Securities Company Limited | 2019 | 601788.SH | East China | 1.99E+04 | 3.24E+09 | 5.68E+08 | 1.33E+09 | 1.11E+08 | 2.18E+08 | 5.47E+09 | 3.64 |
| Everbright Securities Company Limited | 2020 | 601788.SH | East China | 2.31E+04 | 3.58E+09 | 2.33E+09 | 1.45E+09 | 1.15E+08 | 2.21E+08 | 7.70E+09 | 3.00 |
| Everbright Securities Company Limited | 2021 | 601788.SH | East China | 1.88E+04 | 4.02E+09 | 3.48E+09 | 2.85E+09 | 1.29E+08 | 1.11E+08 | 1.06E+10 | 1.77 |
| Everbright Securities Company Limited | 2022 | 601788.SH | East China | 1.10E+04 | 4.04E+09 | 3.19E+09 | 3.09E+09 | 1.52E+08 | 1.28E+08 | 1.06E+10 | 1.04 |
| Shanghai Rural Commercial Bank Co., Ltd. | 2022 | 601825.SH | East China | 4.34E+04 | 4.96E+09 | 1.10E+10 | 5.24E+09 | 5.49E+08 | 1.16E+08 | 2.18E+10 | 1.99 |
| China Zheshang Bank Co.,Ltd. | 2019 | 601916.SH | East China | 1.95E+04 | 7.81E+09 | 1.29E+10 | 6.91E+09 | 0.00E+00 | 7.00E+07 | 2.77E+10 | 0.71 |
| China Zheshang Bank Co.,Ltd. | 2020 | 601916.SH | East China | 1.96E+04 | 7.76E+09 | 1.23E+10 | 8.80E+09 | 1.56E+09 | 0.00E+00 | 3.04E+10 | 0.64 |
| China Zheshang Bank Co.,Ltd. | 2021 | 601916.SH | East China | 1.68E+04 | 8.78E+09 | 1.26E+10 | 1.05E+10 | 1.68E+09 | 0.00E+00 | 3.36E+10 | 0.50 |
| China Zheshang Bank Co.,Ltd. | 2022 | 601916.SH | East China | 4.64E+04 | 1.04E+10 | 1.36E+10 | 1.12E+10 | 1.86E+09 | 0.00E+00 | 3.71E+10 | 1.25 |
| Nanjing Securities Co.,Ltd. | 2022 | 601990.SH | East China | 9.96E+03 | 9.04E+08 | 6.46E+08 | 2.96E+08 | 6.04E+07 | 2.54E+07 | 1.93E+09 | 5.16 |
| Sealand Securities Co., Ltd. | 2021 | 000750.SZ | South China | 1.69E+03 | 1.46E+09 | 7.65E+08 | 5.64E+08 | 7.30E+07 | 5.86E+07 | 2.92E+09 | 0.58 |
| Sealand Securities Co., Ltd. | 2022 | 000750.SZ | South China | 4.62E+03 | 1.59E+09 | 2.49E+08 | 3.22E+08 | 7.39E+07 | 6.32E+07 | 2.30E+09 | 2.01 |
| Sealand Securities Co., Ltd. | 2023 | 000750.SZ | South China | 2.77E+03 | 1.50E+09 | 3.27E+08 | 2.91E+08 | 8.20E+07 | 7.43E+07 | 2.27E+09 | 1.22 |
| Gf Securities Co.,Ltd | 2017 | 000776.SZ | South China | 1.92E+04 | 6.83E+09 | 8.60E+09 | 3.66E+09 | 1.74E+08 | 9.62E+07 | 1.94E+10 | 0.99 |
| Gf Securities Co.,Ltd | 2018 | 000776.SZ | South China | 1.88E+04 | 6.82E+09 | 4.30E+09 | 3.41E+09 | 1.89E+08 | 1.08E+08 | 1.48E+10 | 1.27 |
| Gf Securities Co.,Ltd | 2019 | 000776.SZ | South China | 1.86E+04 | 5.42E+09 | 7.54E+09 | 4.01E+09 | 2.73E+08 | 1.09E+08 | 1.73E+10 | 1.07 |
| Gf Securities Co.,Ltd | 2022 | 000776.SZ | South China | 1.75E+04 | 9.03E+09 | 7.93E+09 | 4.49E+09 | 3.15E+08 | 1.52E+08 | 2.19E+10 | 0.80 |
| Guosen Securities Co.,Ltd. | 2022 | 002736.SZ | South China | 2.93E+04 | 6.03E+09 | 6.09E+09 | 2.03E+09 | 4.68E+08 | 9.24E+07 | 1.47E+10 | 1.99 |
| First Capital Securities Co., Ltd. | 2021 | 002797.SZ | South China | 5.35E+03 | 1.33E+09 | 7.45E+08 | 4.13E+08 | 4.14E+07 | 5.82E+07 | 2.58E+09 | 2.07 |
| First Capital Securities Co., Ltd. | 2022 | 002797.SZ | South China | 3.94E+03 | 1.39E+09 | 4.01E+08 | 2.88E+08 | 4.99E+07 | 7.43E+07 | 2.20E+09 | 1.79 |
| China Great Wall Securities Co.,Ltd. | 2022 | 002939.SZ | South China | 1.73E+03 | 1.74E+09 | 8.99E+08 | 3.71E+08 | 3.89E+07 | 3.36E+07 | 3.08E+09 | 0.56 |
| CITIC Securities Company Limited | 2021 | 600030.SH | South China | 2.76E+04 | 1.89E+10 | 2.31E+10 | 1.34E+10 | 1.27E+09 | 2.71E+08 | 5.69E+10 | 0.48 |
| China Merchants Bank Co., Ltd. | 2021 | 600036.SH | South China | 2.43E+05 | 5.44E+10 | 1.20E+11 | 5.69E+10 | 8.12E+09 | 1.34E+09 | 2.41E+11 | 1.01 |
| China Merchants Securities Co., Ltd | 2021 | 600999.SH | South China | 1.57E+04 | 7.15E+09 | 1.16E+10 | 3.74E+09 | 5.07E+08 | 1.58E+07 | 2.31E+10 | 0.68 |
| Ping An Insurance (Group) Company of China, Ltd. | 2017 | 601318.SH | South China | 1.73E+05 | 5.80E+10 | 8.91E+10 | 6.52E+10 | 4.65E+09 | 2.29E+09 | 2.19E+11 | 0.79 |
| Ping An Insurance (Group) Company of China, Ltd. | 2018 | 601318.SH | South  China | 1.98E+05 | 6.65E+10 | 1.07E+11 | 6.96E+10 | 5.36E+09 | 2.79E+09 | 2.52E+11 | 0.79 |
| Bank of Zhengzhou Co., Ltd. | 2019 | 002936.SZ | Central China | 6.93E+03 | 1.90E+09 | 3.29E+09 | 1.90E+09 | 4.75E+08 | 0.00E+00 | 7.56E+09 | 0.92 |
| Bank of Zhengzhou Co., Ltd. | 2020 | 002936.SZ | Central China | 6.74E+03 | 1.96E+09 | 3.17E+09 | 2.41E+09 | 4.40E+08 | 0.00E+00 | 7.98E+09 | 0.84 |
| Bank of Zhengzhou Co., Ltd. | 2021 | 002936.SZ | Central China | 6.94E+03 | 2.00E+09 | 3.23E+09 | 2.37E+09 | 4.54E+08 | 0.00E+00 | 8.05E+09 | 0.86 |
| Bank of Zhengzhou Co., Ltd. | 2022 | 002936.SZ | Central China | 7.57E+03 | 2.01E+09 | 2.42E+09 | 2.15E+09 | 4.68E+08 | 0.00E+00 | 7.05E+09 | 1.07 |
| Minmetals Capital Company Limited | 2022 | 600390.SH | Central China | 2.87E+03 | 1.95E+09 | 2.53E+09 | 3.65E+09 | 5.96E+07 | 1.01E+08 | 8.28E+09 | 0.35 |
| Tianfeng Securities Co.,Ltd. | 2020 | 601162.SH | Central China | 1.01E+03 | 1.64E+09 | 4.55E+08 | 4.32E+08 | 3.25E+07 | 4.72E+07 | 2.61E+09 | 0.39 |
| Tianfeng Securities Co.,Ltd. | 2021 | 601162.SH | Central China | 1.22E+03 | 2.06E+09 | 5.86E+08 | 3.66E+08 | 4.68E+07 | 4.28E+07 | 3.11E+09 | 0.39 |
| Tianfeng Securities Co.,Ltd. | 2022 | 601162.SH | Central China | 7.87E+02 | 2.17E+09 | -1.51E+09 | 7.59E+08 | 6.08E+07 | 3.89E+07 | 1.52E+09 | 0.52 |
| Central China Securities Co., Ltd. | 2021 | 601375.SH | Central China | 1.19E+03 | 1.14E+09 | 5.13E+08 | 4.72E+08 | 2.99E+07 | 3.79E+07 | 2.19E+09 | 0.54 |
| Central China Securities Co., Ltd. | 2022 | 601375.SH | Central China | 9.32E+02 | 1.18E+09 | 1.07E+08 | 2.01E+08 | 3.21E+07 | 4.82E+07 | 1.56E+09 | 0.60 |
| China Securities Co.,Ltd. | 2020 | 601066.SH | North China | 6.83E+03 | 5.17E+09 | 9.51E+09 | 3.70E+09 | 1.21E+08 | 8.39E+07 | 1.86E+10 | 0.37 |
| China Securities Co.,Ltd. | 2021 | 601066.SH | North China | 4.62E+03 | 7.08E+09 | 1.02E+10 | 4.02E+09 | 1.71E+08 | 1.20E+08 | 2.16E+10 | 0.21 |
| China Securities Co.,Ltd. | 2022 | 601066.SH | North China | 1.02E+04 | 8.22E+09 | 7.51E+09 | 4.03E+09 | 2.35E+08 | 1.66E+08 | 2.02E+10 | 0.51 |
| Bank of Beijing Co., Ltd. | 2022 | 601169.SH | North China | 6.47E+04 | 8.79E+09 | 2.48E+10 | 1.25E+10 | 2.43E+09 | 7.03E+08 | 4.92E+10 | 1.32 |
| Agricultural Bank of China Limited | 2017 | 601288.SH | North China | 3.05E+04 | 1.13E+11 | 1.93E+11 | 6.14E+10 | 1.55E+10 | 1.57E+09 | 3.85E+11 | 0.08 |
| Agricultural Bank of China Limited | 2021 | 601288.SH | North China | 2.58E+05 | 1.35E+11 | 2.41E+11 | 9.92E+10 | 1.75E+10 | 1.88E+09 | 4.95E+11 | 0.52 |
| Agricultural Bank of China Limited | 2022 | 601288.SH | North China | 2.15E+06 | 1.36E+11 | 2.59E+11 | 1.14E+11 | 1.79E+10 | 2.45E+09 | 5.29E+11 | 4.07 |
| New China Life Insurance Company Ltd. | 2021 | 601336.SH | North China | 1.90E+04 | 8.99E+09 | 1.49E+10 | 3.19E+09 | 7.40E+08 | 3.13E+08 | 2.82E+10 | 0.67 |
| Industrial and Commercial Bank of China Limited | 2016 | 601398.SH | North China | 5.36E+04 | 1.12E+11 | 2.78E+11 | 1.43E+11 | 1.87E+10 | 0.00E+00 | 5.52E+11 | 0.10 |
| China Life Insurance Company Limited | 2017 | 601628.SH | North China | 1.71E+04 | 1.90E+10 | 3.23E+10 | 8.32E+09 | 1.79E+09 | 2.55E+08 | 6.16E+10 | 0.28 |
| China Life Insurance Company Limited | 2018 | 601628.SH | North China | 1.18E+05 | 2.18E+10 | 1.14E+10 | 1.31E+10 | 2.11E+09 | 2.69E+08 | 4.87E+10 | 2.42 |
| China Life Insurance Company Limited | 2019 | 601628.SH | North China | 1.42E+05 | 2.41E+10 | 5.83E+10 | 1.18E+10 | 2.53E+09 | 3.14E+08 | 9.71E+10 | 1.46 |
| China Life Insurance Company Limited | 2020 | 601628.SH | North China | 1.50E+06 | 2.35E+10 | 5.03E+10 | 8.32E+09 | 2.91E+09 | 3.23E+08 | 8.53E+10 | 17.53 |
| China Life Insurance Company Limited | 2021 | 601628.SH | North China | 1.06E+06 | 2.41E+10 | 5.09E+10 | 1.21E+10 | 3.15E+09 | 3.39E+08 | 9.06E+10 | 11.73 |
| China Life Insurance Company Limited | 2022 | 601628.SH | North China | 7.08E+05 | 2.63E+10 | 3.21E+10 | 6.23E+09 | 3.43E+09 | 3.60E+08 | 6.84E+10 | 10.35 |
| Postal Savings Bank of China Co.,Ltd. | 2020 | 601658.SH | North China | 1.34E+05 | 4.99E+10 | 6.42E+10 | 2.18E+10 | 7.13E+09 | 9.52E+08 | 1.44E+11 | 0.93 |
| China Galaxy Securities Co.,Ltd. | 2019 | 601881.SH | North China | 2.44E+04 | 4.02E+09 | 5.23E+09 | 2.31E+09 | 8.98E+07 | 6.67E+07 | 1.17E+10 | 2.08 |
| China Galaxy Securities Co.,Ltd. | 2020 | 601881.SH | North China | 2.52E+04 | 4.97E+09 | 7.24E+09 | 2.49E+09 | 1.11E+08 | 8.42E+07 | 1.49E+10 | 1.69 |
| China Galaxy Securities Co.,Ltd. | 2021 | 601881.SH | North China | 1.78E+04 | 5.88E+09 | 1.04E+10 | 3.51E+09 | 1.43E+08 | 1.07E+08 | 2.01E+10 | 0.89 |
| China Galaxy Securities Co.,Ltd. | 2022 | 601881.SH | North China | 1.90E+04 | 6.67E+09 | 7.76E+09 | 2.23E+09 | 2.04E+08 | 1.64E+08 | 1.70E+10 | 1.12 |
| China Construction Bank Corporation | 2016 | 601939.SH | North China | 6.93E+04 | 9.31E+10 | 2.31E+11 | 1.03E+11 | 1.60E+10 | 0.00E+00 | 4.43E+11 | 0.16 |
| Bank of China Limited | 2018 | 601988.SH | North China | 7.86E+04 | 8.36E+10 | 1.80E+11 | 5.71E+10 | 1.34E+10 | 3.64E+09 | 3.38E+11 | 0.23 |
| Bank of China Limited | 2019 | 601988.SH | North China | 7.58E+04 | 8.87E+10 | 1.87E+11 | 5.74E+10 | 2.11E+10 | 4.22E+09 | 3.59E+11 | 0.21 |
| Bank of China Limited | 2020 | 601988.SH | North China | 7.58E+04 | 8.88E+10 | 1.93E+11 | 9.25E+10 | 2.24E+10 | 5.07E+09 | 4.02E+11 | 0.19 |
| Bank of China Limited | 2021 | 601988.SH | North China | 1.53E+06 | 9.40E+10 | 2.17E+11 | 9.49E+10 | 2.24E+10 | 6.36E+09 | 4.34E+11 | 3.53 |
| Bank of China Limited | 2022 | 601988.SH | North China | 1.54E+06 | 9.60E+10 | 2.27E+11 | 8.62E+10 | 2.24E+10 | 6.76E+09 | 4.39E+11 | 3.50 |
| China International Capital Corporation Limited | 2020 | 601995.SH | North China | 3.11E+04 | 7.72E+09 | 7.21E+09 | 3.06E+09 | 2.08E+08 | 1.46E+08 | 1.84E+10 | 1.69 |
| China International Capital Corporation Limited | 2021 | 601995.SH | North China | 2.06E+04 | 1.08E+10 | 1.08E+10 | 3.24E+09 | 2.62E+08 | 1.55E+08 | 2.52E+10 | 0.82 |
| China International Capital Corporation Limited | 2022 | 601995.SH | North China | 1.92E+04 | 1.42E+10 | 7.60E+09 | 3.14E+09 | 3.24E+08 | 2.72E+08 | 2.56E+10 | 0.75 |
| China Citic Bank Corporation Limited | 2023 | 601998.SH | North China | 1.53E+05 | 3.76E+10 | 6.70E+10 | 3.11E+10 | 4.90E+09 | 0.00E+00 | 1.41E+11 | 1.09 |
| Northeast Securities Co.,Ltd. | 2022 | 000686.SZ | Northeast | 9.07E+03 | 1.93E+09 | 2.31E+08 | 6.97E+08 | 7.31E+07 | 5.10E+07 | 2.99E+09 | 3.04 |
| AVIC Industry-Finance Holdings Co.,Ltd. | 2020 | 600705.SH | Northeast | 3.62E+03 | 1.87E+09 | 3.27E+09 | 4.85E+09 | 1.10E+09 | 2.11E+07 | 1.11E+10 | 0.33 |
| Shenwan Hongyuan Group Co.,Ltd. | 2020 | 000166.SZ | Northwest | 8.99E+03 | 5.40E+09 | 7.77E+09 | 2.57E+09 | 1.61E+08 | 8.06E+07 | 1.60E+10 | 0.56 |
| Shenwan Hongyuan Group Co.,Ltd. | 2021 | 000166.SZ | Northwest | 1.12E+04 | 7.19E+09 | 9.40E+09 | 3.86E+09 | 1.70E+08 | 9.97E+07 | 2.07E+10 | 0.54 |
| Shenwan Hongyuan Group Co.,Ltd. | 2022 | 000166.SZ | Northwest | 1.30E+04 | 8.00E+09 | 2.79E+09 | 3.16E+09 | 1.90E+08 | 1.34E+08 | 1.43E+10 | 0.91 |
| Cnpc Capital Company Limited | 2022 | 000617.SZ | Northwest | 5.09E+03 | 2.11E+09 | 4.92E+09 | 4.61E+09 | 6.20E+08 | 8.18E+07 | 1.23E+10 | 0.41 |
| Sinolink Securities Co., Ltd. | 2021 | 600109.SH | Southwest | 1.06E+04 | 2.81E+09 | 2.32E+09 | 1.06E+09 | 4.98E+07 | 4.09E+07 | 6.28E+09 | 1.69 |
| Sinolink Securities Co., Ltd. | 2022 | 600109.SH | Southwest | 3.90E+03 | 3.29E+09 | 1.20E+09 | 1.13E+09 | 5.65E+07 | 4.70E+07 | 5.71E+09 | 0.68 |
| Southwest Securities Co.,Ltd. | 2022 | 600369.SH | Southwest | 3.38E+03 | 1.22E+09 | 3.09E+08 | 3.21E+08 | 8.62E+07 | 3.94E+07 | 1.98E+09 | 1.71 |

**Table S3 The total CO_2_ emissions of 31 provinces in 2012, 2015, 2017 and 2020 (unit: 10^10^ g CO_2_-eq).**

| Provinces | Acronyms | 2012 | 2015 | 2017 | 2020 |
| --- | --- | --- | --- | --- | --- |
| 1.Beijing | 1.BJ | 59.61 | 92.25 | 109.39 | 168.89 |
| 2.Tianjin | 2.TJ | 23.53 | 37.67 | 45.86 | 48.33 |
| 3.Hebei | 3.HB | 21.47 | 34.80 | 48.25 | 61.08 |
| 4.Shanxi | 4.SX | 15.03 | 26.80 | 31.02 | 28.38 |
| 5.Nei Mongol | 5.NMG | 11.80 | 19.48 | 25.84 | 20.89 |
| 6.Liaoning | 6.LN | 10.64 | 20.51 | 21.56 | 23.08 |
| 7.Jilin | 7.JL | 2.68 | 6.20 | 7.79 | 9.89 |
| 8.Heilongjiang | 8.HLJ | 5.32 | 9.30 | 10.23 | 11.55 |
| 9.Shanghai | 9.SH | 39.70 | 67.45 | 86.37 | 116.11 |
| 10.Jiangsu | 10.JS | 50.82 | 85.92 | 109.91 | 136.19 |
| 11.Zhejiang | 11.ZJ | 44.75 | 47.36 | 57.24 | 90.58 |
| 12.Anhui | 12.AH | 10.01 | 20.12 | 26.95 | 41.38 |
| 13.Fujian | 13.FJ | 16.45 | 27.24 | 33.30 | 55.39 |
| 14.Jiangxi | 14.JX | 6.69 | 14.54 | 17.94 | 29.30 |
| 15.Shandong | 15.SD | 31.37 | 48.52 | 59.16 | 74.00 |
| 16.Henan | 16.HN | 6.57 | 12.90 | 16.26 | 19.16 |
| 17.Hubei | 17.HB | 5.64 | 12.01 | 17.12 | 19.62 |
| 18.Hunan | 18.HN | 3.76 | 7.16 | 10.44 | 13.78 |
| 19.Guangdong | 19.GD | 35.88 | 65.13 | 77.53 | 112.08 |
| 20.Guangxi | 20.GX | 6.48 | 11.52 | 14.41 | 18.08 |
| 21.Hainan | 21.HN | 1.48 | 2.75 | 3.50 | 4.50 |
| 22.Chongqing | 22.CQ | 10.05 | 15.47 | 19.90 | 24.28 |
| 23.Sichuan | 23.SC | 14.30 | 24.16 | 35.15 | 37.04 |
| 24.Guizhou | 24.GZ | 4.01 | 6.66 | 8.65 | 12.53 |
| 25.Yunnan | 25.YN | 5.94 | 10.77 | 13.11 | 16.46 |
| 26.Xizang | 26.XZ | 0.35 | 0.75 | 1.21 | 1.52 |
| 27.Shaanxi | 27.SX | 6.05 | 11.88 | 14.27 | 19.98 |
| 28.Gansu | 28.GS | 2.02 | 4.86 | 6.07 | 9.85 |
| 29.Qinghai | 29.QH | 0.92 | 2.42 | 3.01 | 2.89 |
| 30.Ningxia | 30.NX | 1.84 | 2.81 | 3.45 | 3.52 |
| 31.Xinjiang | 31.XJ | 3.95 | 6.19 | 6.84 | 11.92 |

**Table S4 The inflow and outflow of CO_2_ emissions of 31 provinces in 2012, 2015, 2017, and 2020 (unit: 10^10^ g CO_2_-eq).**

|  | Inflow | | | | Outflow | | | |
| --- | --- | --- | --- | --- | --- | --- | --- | --- |
| Provinces | 2012 | 2015 | 2017 | 2020 | 2012 | 2015 | 2017 | 2020 |
| 1.Beijing | 130.74 | 199.35 | 217.29 | 269.22 | 173.87 | 282.78 | 240.68 | 280.76 |
| 2.Tianjin | 70.24 | 120.75 | 75.72 | 79.01 | 86.64 | 151.91 | 88.31 | 89.31 |
| 3.Hebei | 104.40 | 144.11 | 81.90 | 68.39 | 86.37 | 129.17 | 86.14 | 86.00 |
| 4.Shanxi | 49.44 | 74.82 | 55.19 | 58.64 | 67.49 | 116.51 | 68.37 | 68.90 |
| 5.Nei Mongol | 41.20 | 56.73 | 34.78 | 30.93 | 48.34 | 82.45 | 28.84 | 17.14 |
| 6.Liaoning | 48.63 | 65.50 | 35.00 | 26.82 | 40.87 | 77.14 | 33.05 | 28.35 |
| 7.Jilin | 21.22 | 34.67 | 19.23 | 18.04 | 9.49 | 22.79 | 19.57 | 25.62 |
| 8.Heilongjiang | 19.82 | 28.21 | 17.78 | 16.56 | 17.57 | 33.08 | 18.93 | 19.75 |
| 9.Shanghai | 101.13 | 171.94 | 172.84 | 215.88 | 127.45 | 252.42 | 179.65 | 210.96 |
| 10.Jiangsu | 167.49 | 297.94 | 201.95 | 222.62 | 160.37 | 303.91 | 181.57 | 194.30 |
| 11.Zhejiang | 158.82 | 245.45 | 127.49 | 108.69 | 155.59 | 157.54 | 124.51 | 105.86 |
| 12.Anhui | 49.10 | 140.38 | 66.38 | 76.75 | 35.41 | 76.49 | 55.53 | 67.59 |
| 13.Fujian | 60.33 | 113.23 | 53.28 | 49.06 | 59.94 | 128.01 | 57.62 | 56.23 |
| 14.Jiangxi | 28.75 | 49.29 | 29.89 | 30.57 | 25.29 | 50.69 | 26.27 | 26.86 |
| 15.Shandong | 128.26 | 215.34 | 72.26 | 38.66 | 128.34 | 228.77 | 62.21 | 22.54 |
| 16.Henan | 39.89 | 82.23 | 48.39 | 53.48 | 24.12 | 49.38 | 30.96 | 35.06 |
| 17.Hubei | 24.08 | 51.32 | 32.45 | 37.48 | 17.96 | 39.01 | 33.62 | 43.01 |
| 18.Hunan | 15.91 | 34.51 | 18.30 | 19.74 | 11.62 | 22.60 | 15.86 | 18.40 |
| 19.Guangdong | 114.29 | 247.62 | 137.31 | 151.13 | 87.34 | 192.25 | 146.72 | 182.34 |
| 20.Guangxi | 22.01 | 46.76 | 25.16 | 27.06 | 20.43 | 41.34 | 24.81 | 27.44 |
| 21.Hainan | 6.67 | 11.36 | 7.04 | 7.26 | 5.46 | 11.40 | 4.02 | 3.16 |
| 22.Chongqing | 27.68 | 67.09 | 33.68 | 37.28 | 35.93 | 53.18 | 30.93 | 27.93 |
| 23.Sichuan | 50.20 | 95.71 | 61.96 | 69.01 | 50.76 | 84.90 | 68.47 | 79.09 |
| 24.Guizhou | 14.92 | 30.23 | 14.62 | 14.43 | 14.44 | 24.54 | 13.09 | 12.28 |
| 25.Yunnan | 18.47 | 35.44 | 22.16 | 24.37 | 20.60 | 35.19 | 21.53 | 22.09 |
| 26.Xizang | 1.07 | 1.49 | 2.75 | 3.75 | 0.74 | 2.16 | 3.16 | 4.61 |
| 27.Shaanxi | 24.31 | 42.51 | 29.27 | 32.24 | 25.21 | 46.77 | 28.78 | 30.92 |
| 28.Gansu | 9.83 | 18.10 | 12.54 | 14.16 | 8.40 | 18.76 | 14.06 | 17.46 |
| 29.Qinghai | 3.66 | 7.32 | 6.35 | 7.96 | 3.85 | 9.69 | 6.01 | 7.31 |
| 30.Ningxia | 6.79 | 11.68 | 6.96 | 7.06 | 8.23 | 13.00 | 6.10 | 4.82 |
| 31.Xinjiang | 14.70 | 20.48 | 15.50 | 15.98 | 15.93 | 23.76 | 16.04 | 16.12 |

**Table S5 The** **net embodied CO_2_ emissions and density (NCD) from interregional trade. Unit of net embodied CO_2_ emissions: 10^10^ g CO_2_-eq; Unit of NCD: g CO_2_-eq/10^2^yuan.**

|  | Net embodied CO_2_ emissions | | | | NCD | | | |
| --- | --- | --- | --- | --- | --- | --- | --- | --- |
| Provinces | 2012 | 2015 | 2017 | 2020 | 2012 | 2015 | 2017 | 2020 |
| 1.Beijing | -43.13 | -83.43 | -23.39 | -11.55 | -170.02 | -212.49 | -50.25 | -16.07 |
| 2.Tianjin | -16.40 | -31.16 | -12.59 | -10.30 | -163.79 | -194.33 | -64.49 | -50.07 |
| 3.Hebei | 18.03 | 14.94 | -4.24 | -17.61 | 197.39 | 100.90 | -20.65 | -67.73 |
| 4.Shanxi | -18.05 | -41.69 | -13.18 | -10.26 | -282.15 | -365.54 | -99.84 | -84.95 |
| 5.Nei Mongol | -7.14 | -25.72 | 5.94 | 13.79 | -142.21 | -310.19 | 54.04 | 155.18 |
| 6.Liaoning | 7.76 | -11.64 | 1.95 | -1.54 | 80.08 | -62.25 | 9.92 | -7.32 |
| 7.Jilin | 11.73 | 11.88 | -0.34 | -7.59 | 479.50 | 210.21 | -4.82 | -84.17 |
| 8.Heilongjiang | 2.26 | -4.87 | -1.15 | -3.19 | 46.55 | -57.46 | -12.30 | -30.30 |
| 9.Shanghai | -26.33 | -80.48 | -6.80 | 4.91 | -107.44 | -193.33 | -12.76 | 6.86 |
| 10.Jiangsu | 7.12 | -5.97 | 20.37 | 28.33 | 22.70 | -11.26 | 30.03 | 33.70 |
| 11.Zhejiang | 3.23 | 87.91 | 2.98 | 2.83 | 11.69 | 300.76 | 8.44 | 5.07 |
| 12.Anhui | 13.69 | 63.89 | 10.86 | 9.16 | 221.69 | 514.48 | 65.27 | 35.86 |
| 13.Fujian | 0.39 | -14.79 | -4.34 | -7.18 | 3.84 | -87.94 | -21.12 | -21.00 |
| 14.Jiangxi | 3.45 | -1.40 | 3.62 | 3.71 | 83.63 | -15.55 | 32.66 | 20.53 |
| 15.Shandong | -0.08 | -13.43 | 10.05 | 16.12 | -0.40 | -44.84 | 27.52 | 35.30 |
| 16.Henan | 15.77 | 32.85 | 17.43 | 18.43 | 155.56 | 165.01 | 69.46 | 62.34 |
| 17.Hubei | 6.12 | 12.31 | -1.17 | -5.54 | 70.26 | 66.42 | -4.42 | -18.29 |
| 18.Hunan | 4.29 | 11.91 | 2.44 | 1.33 | 74.00 | 107.82 | 15.16 | 6.27 |
| 19.Guangdong | 26.95 | 55.36 | -9.40 | -31.22 | 84.97 | 96.16 | -13.72 | -31.51 |
| 20.Guangxi | 1.57 | 5.42 | 0.35 | -0.39 | 27.47 | 53.22 | 2.74 | -2.41 |
| 21.Hainan | 1.22 | -0.04 | 3.02 | 4.10 | 93.16 | -1.63 | 97.73 | 103.04 |
| 22.Chongqing | -8.25 | 13.91 | 2.75 | 9.35 | -90.07 | 98.66 | 15.16 | 42.25 |
| 23.Sichuan | -0.56 | 10.82 | -6.51 | -10.08 | -4.30 | 49.11 | -20.32 | -29.86 |
| 24.Guizhou | 0.48 | 5.69 | 1.53 | 2.15 | 13.17 | 93.77 | 19.37 | 18.86 |
| 25.Yunnan | -2.13 | 0.26 | 0.63 | 2.29 | -39.38 | 2.62 | 5.27 | 15.24 |
| 26.Xizang | 0.33 | -0.67 | -0.42 | -0.86 | 102.85 | -98.00 | -37.76 | -62.33 |
| 27.Shaanxi | -0.90 | -4.27 | 0.49 | 1.32 | -16.40 | -39.41 | 3.75 | 7.27 |
| 28.Gansu | 1.43 | -0.66 | -1.53 | -3.30 | 77.29 | -14.81 | -27.56 | -36.73 |
| 29.Qinghai | -0.20 | -2.37 | 0.33 | 0.65 | -23.53 | -107.42 | 12.07 | 24.59 |
| 30.Ningxia | -1.43 | -1.32 | 0.86 | 2.23 | -85.65 | -51.45 | 27.25 | 69.54 |
| 31.Xinjiang | -1.22 | -3.27 | -0.54 | -0.13 | -33.96 | -58.05 | -8.69 | -1.22 |
